# The role of leptomeningeal collaterals in redistributing blood flow during stroke

**DOI:** 10.1371/journal.pcbi.1011496

**Published:** 2023-10-23

**Authors:** Robert Epp, Chaim Glück, Nadine Felizitas Binder, Mohamad El Amki, Bruno Weber, Susanne Wegener, Patrick Jenny, Franca Schmid

**Affiliations:** 1 Institute of Fluid Dynamics, ETH Zurich, Zurich, Switzerland; 2 Institute of Pharmacology and Toxicology, University of Zurich, Zurich, Switzerland; 3 Deptartment of Neurology, University Hospital Zurich and University of Zurich, Zurich, Switzerland; 4 ARTORG Center for Biomedical Engineering Research, University of Bern, Bern, Switzerland; University of Arizona, UNITED STATES

## Abstract

Leptomeningeal collaterals (LMCs) connect the main cerebral arteries and provide alternative pathways for blood flow during ischaemic stroke. This is beneficial for reducing infarct size and reperfusion success after treatment. However, a better understanding of how LMCs affect blood flow distribution is indispensable to improve therapeutic strategies. Here, we present a novel *in silico* approach that incorporates case-specific *in vivo* data into a computational model to simulate blood flow in large semi-realistic microvascular networks from two different mouse strains, characterised by having many and almost no LMCs between middle and anterior cerebral artery (MCA, ACA) territories. This framework is unique because our simulations are directly aligned with *in vivo* data. Moreover, it allows us to analyse perfusion characteristics quantitatively across all vessel types and for networks with no, few and many LMCs. We show that the occlusion of the MCA directly caused a redistribution of blood that was characterised by increased flow in LMCs. Interestingly, the improved perfusion of MCA-sided microvessels after dilating LMCs came at the cost of a reduced blood supply in other brain areas. This effect was enhanced in regions close to the watershed line and when the number of LMCs was increased. Additional dilations of surface and penetrating arteries after stroke improved perfusion across the entire vasculature and partially recovered flow in the obstructed region, especially in networks with many LMCs, which further underlines the role of LMCs during stroke.

## Author summary

Cerebral ischaemic strokes are a leading cause of death and disability worldwide. Among other factors, the outcome of stroke treatment is determined by the existence and extent of collateral flow paths, which sustain residual blood supply to the obstructed brain region. To improve therapeutic strategies and to reduce reperfusion injuries during treatment, an in-depth understanding of the role of collaterals for maintaining blood supply is indispensable. We performed numerical simulations to quantify how leptomeningeal collaterals impact blood flow redistribution in response to middle cerebral artery occlusion. Our studies have the unique feature that they are consistent with the topology of case-specific pial arterial networks from mouse brains and aligned with sparse *in vivo* blood flow measurements. This allows the valuable joint interpretation of numerical studies and *in vivo* experiments. We observed that maintaining perfusion to the obstructed region comes at the cost of reduced blood supply in other areas. Moreover, dilation of arterial vessels improved perfusion in the entire vasculature. Importantly, flow changes vary significantly across and even within vessel types, which underlines the benefits of numerical models with single vessel resolution. Taken together, our framework establishes a strong link between experimental and numerical studies necessary to advance our understanding of perfusion changes in response to stroke and after clot removal.

## Introduction

Due to the limited energy storage of the brain, maintaining a robust oxygen and nutrient supply is crucial. During healthy conditions, the interconnected network of microvascular blood vessels [1–3] sustains blood flow to all brain areas and regulates flow in response to local changes in neuronal activity [4–6]. However, during ischaemic stroke blood supply to specific brain regions is reduced drastically by a clot obstructing large arterial vessels. This typically causes tissue damage, which often results in permanent disability or even death [7].

Leptomeningeal anastomoses or collaterals (LMCs) are blood vessels connecting branches of major feeding arteries at the cortical surface of the brain [8, 9], e.g. the middle (MCA) and the anterior (ACA) cerebral arteries. Due to their relatively low flow velocity [10] and small vessel diameters, LMCs are often described as being “almost dormant” during healthy physiological conditions. However, during stroke LMCs dilate [11–14] and provide alternative routes for blood to partially maintain perfusion in under-supplied brain regions [7].

Current treatments for stroke include the removal of the clot by either thrombolysis with recombinant tissue plasminogen activator (rt-PA) or mechanical thrombectomy [7]. Among other factors, the outcome of stroke treatment is determined by the existence and extent of leptomeningeal collaterals of individual patients [7]. However, even after successful recanalisation of the occluded vessel, some brain regions may remain unperfused, e.g. due to microvascular obstructions by neutrophils [15], secondary occlusions caused by fragments of the original clot [16] or constrictions of downstream vessels [17]. In order to improve therapeutic strategies, an in-depth understanding of how LMCs redistribute flow during stroke across vessel types is essential [8, 16].

Studies in mice showed that the diameter and number of LMCs vary for different strains [11, 12], but also between individual animals [18]. This directly impacts the infarct volume and therewith the overall outcome after stroke [12, 14, 19–21]. Furthermore, it has been suggested that functional activation of the cortex can offer protection from stroke, possibly by re-routing flow over collateral flow paths [22, 23]. While experimental studies generally agree that the presence of many large LMCs reduces the infarct size after stroke [12, 14, 19, 20], relatively little is known on how LMCs precisely redistribute flow at the level of individual vessels and across the entire vasculature. Even though scanning speeds for two-photon microscopy (2PM) line scans are increasing [24, 25], generally 2PM studies are limited to quantifying flow in a few vessels [13, 26–28]. Moreover, data on perfusion changes in all vessel types, e.g. arteries and capillaries, and over the entire depth of the cortex is usually not available. Other measurement techniques such as functional magnetic resonance (fMRI) [29, 30] or laser speckle contrast imaging (LSI) [15, 31] provide results on a more global scale. However, their resolution is either very coarse (fMRI) or the results are difficult to interpret quantitatively (LSI) [31]. More-advanced high-resolution tomographic imaging methods [32–35] have the potential to quantify flow in large brain regions at single vessel resolution. While these methods are very promising for the future, they are not yet widely used in the field.

In contrast to *in vivo* studies, the numerical simulations employed here offer the advantage that flow values for the entire vasculature are obtained [36–39]. Furthermore, individual vessel diameters or even the network topology can be adjusted to evaluate the isolated impact of these changes on overall perfusion characteristics [40–44]. This is challenging *in vivo*, where localised vascular modifications may trigger a series of changes within large areas of the brain. Thus, *in silico* studies are a convenient tool to provide novel insights on the role of LMCs on flow redistribution during stroke across all vessel types.

To simulate blood flow, a network representation of the vasculature is required. Generally, the acquisition, segmentation and vectorisation of large realistic microvascular networks with thousands of vessels is challenging, especially if complete connectivity and accurate capillary diameters are required, as it is the case for blood flow simulations [3, 36, 37]. Nonetheless, recent advances in imaging and data processing allow the *ex vivo* mapping of the entire mouse brain vasculature [45–48]. However, these networks have not been used in simulation studies yet, and a direct comparison to *in vivo* flow measurements would be difficult. Alternatively, fully artificial or semi-realistic networks matching realistic characteristics can be used [21, 49, 50].

Here, we present a novel approach that allows us to generate large semi-realistic microvascular networks that are based on case-specific experimental data available from *in vivo* studies. More precisely, our framework combines incomplete pial arterial networks obtained from mice with realistic penetrating trees and an artificial capillary bed to obtain a network that mimics the realistic vasculature. By applying an inverse model the networks are tuned such that they are aligned with *in vivo* two-photon microscopy red blood cell (RBC) velocity measurements [15, 38, 51] from individual subjects and literature. These networks are then used for *in silico* experiments studying changes of blood flow distributions during stroke. While inverse modelling has previously been used to incorporate experimental data into blood flow simulations [52–58], to the best of our knowledge, our framework is the first that tunes network characteristics in large semi-realistic microvascular networks by incorporating sparse data from *in vivo* experiments at arterial level. Importantly, our framework is well-suited to handle both large parameter spaces and a large number of constraints. This strong connection between *in vivo* and *in silico* allows to reduce uncertainties and opens the door for novel interesting research questions.

In the current study we used our novel simulation framework to compare the flow fields during (1) baseline (Base), (2) MCA occlusion (MCAo), (3) after subsequent LMC dilation (MCAo & LMC-dil) and (4) after additionally dilating all arteries (MCAo & LMC/SA/DA-dil) for vascular networks derived from mice with (C57BL/6) and no LMCs (BALB/c). Additionally, we analysed the flow field in modified test cases by adding or removing LMCs. This approach allowed us to study the flow and pressure fields in the entire vasculature during stroke and LMC dilation. Based on that, we provide novel insight on the role of LMCs and comment on the impact of the number of LMCs for the overall perfusion.

## Materials and methods

### Ethics statement

All animal experiments were approved by the local veterinary authorities in Zurich and conformed to the guidelines of the Swiss Animal Protection Law, Veterinary Office, Canton of Zurich (Act of Animal Protection 16 December 2005 and Animal Protection Ordinance 23 April 2008, animal welfare assurance numbers ZH165/19 and ZH224/15).

### In vivo experiments in pial arteries

**Fig 1:**
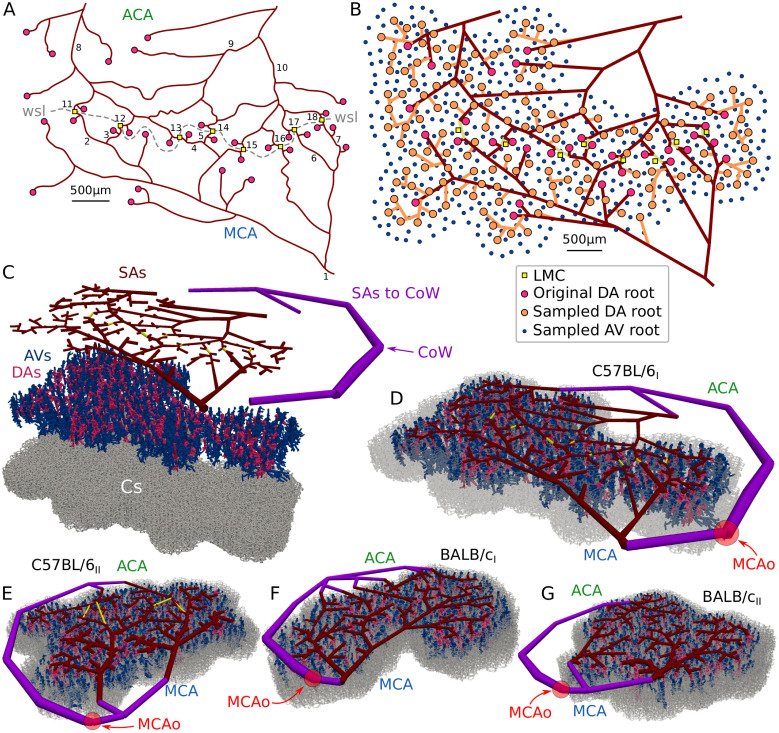
Stepwise generation of large semi-realistic microvascular networks based on incomplete experimental data. (A) Experimentally acquired reconstruction of surface artery (SA) network from a mouse with LMCs (network C57BL/6_I_). The MCA and ACA sided SAs are fed by the respective main feeding arteries, and are connected by LMCs (yellow squares) across the watershed line (wsl). The numbers refer to diameter and RBC velocity measurements obtained in individual SAs (S1 Table). Red dots are used to mark the locations of descending artery (DA) root points. For the reconstructions and measurements of the other three networks, refer to S9 Fig, and S24, S25 and S26 Tables, respectively. (B) Network representing SAs. The original topology is shown with red lines and the additionally added SAs to connect the sampled DA roots (orange dots) are indicated by orange lines. Furthermore, ascending vein (AV) roots (blue dots) are distributed around the DAs. (C) Hierarchical structure of the microvascular network consisting of SAs, DAs, AVs and capillaries (Cs). Additional SAs are added to connect the MCA and ACA sided SAs to a common inflow vertex, i.e., the circle of Willis (CoW). (D-G) Networks C57BL/6_I_ and C57BL/6_II_ with LMCs (D-E) and BALB/c_I_ and BALB/c_II_ without LMCs (F-G). The locations of MCA occlusion (MCAo) are indicated with red circles. The networks are located in the MCA-M4/M5 territory and the MCAo is at the MCA-M2 [61].

Our study builds on *in vivo* data from two different mouse strains characterised by either having many (C57BL/6) or close to no (BALB/c) LMCs between the MCA and ACA territories [11, 12, 20]. *In vivo* two-photon imaging was used to extract the topology and vessel lengths of four pial or surface artery (SA) networks (2x C57BL/6J (JAX: #000664), 2x BALB/cByJ (JAX: #001026)) located at the cortical surface in the whisker and hindlimb area of the cortex (cranial window size 3.5 x 3.5mm^2^, MCA-M4/M5 territory, S10 Fig). Animal surgery for two-photon imaging and blood flow measurements were performed as described earlier [15]. Fig 1A shows a manually traced reconstruction of SAs located at the MCA-ACA watershed line (wsl). The SAs are fed by either MCA or ACA, and for C57BL/6 networks the downstream MCA and ACA sided SAs are connected by LMCs (yellow squares). All visible descending artery (DA) roots, i.e., locations where DAs branch off from the SAs to supply the capillary bed, are marked by red dots. Diameters and RBC velocities were measured by line-scans and were processed with a custom-designed image processing tool box for MATLAB (Cellular and Hemodynamic Image Processing Suite, CHIPS, [59]; R2014b; MathWorks). Vessel diameters at baseline were determined at full width half maximum from a Gaussian fitted intensity profile drawn perpendicular to the vessel axis for a subset of vessels in all networks. Additionally, baseline RBC velocities in selected vessels of one C57BL/6 and one BALB/c network were analysed with the Radon method [60] implemented in CHIPS (see S1 Table for the measurements of the network C57BL/6_I_ in Fig 1A).

### Generation of semi-realistic microvascular networks

To study perfusion characteristics in the entire vasculature, we generated semi-realistic microvascular networks that are based on realistic pial topologies (Fig 1A) but also contain penetrating trees and capillaries. Within this manuscript we use the term descending artery (DA) and ascending vein (AV) to refer to all arterial and venule vessels below the cortical surface, i.e., this includes arterioles and venules. Detecting all DA roots in the area below the cortical window is difficult without acquiring complete z-stacks. Therefore, in a first step, we increased the number of DA roots to match experimental values as observed in high-resolution images of the vasculature obtained by light sheet microscopy [61] (Table A in S1 Appendix). To attain this goal we sampled additional tree locations directly into the SA reconstruction (orange dots in Fig 1B) and connected the novel DA roots to the nearest SAs by introducing new vessels (orange edges in Fig 1B). See S1 Appendix for more details on the refinement algorithm for the pial vasculature. Additionally, root points of venous trees at the cortical surface, i.e., the AV roots, were added such that they are located around DA roots and mimic a rhombic lattice with a DA:AV ratio of 1:3 [3] (blue dots in Fig 1B). Importantly, the resulting pial network (Fig 1B) is still characterised by the overall SA topology of the originally acquired network (Fig 1A). However, the density of DA roots is now consistent with mean values observed in high-resolution experiments. While in the current study a mean DA root density was prescribed for the entire network, the applied sampling approach would also allow to match a spatially varying distribution of penetrating trees, given such data is available from experimental studies.

The refined SA topology (Fig 1B) was used to generate large three-dimensional semi-realistic microvascular networks consisting of pial arteries (SAs), descending arteries (DAs), capillaries (Cs) and ascending veins (AVs) (Fig 1C). When available, *in vivo* diameter measurements were assigned to the refined SA topology (S1 Table). The remaining vessel diameters were estimated by interpolating between the available values. Note that the prescribed vessel diameters are only preliminary and their uncertainties will be reduced in a subsequent step by using inverse modelling. Additional pial arteries (SAs to CoW) were added to connect the MCA and ACA inflows to a common inflow vertex, which represents the vessel offspring at the circle of Willis (CoW). Subsequently, we added realistic penetrating trees (DAs, AVs) and connect them to the pial network at the DA and AV root points. The penetrating trees were sampled from a database previously obtained from the somatosensory cortex of the rat [36, 62], and scaled by a factor of 2/3 to account for size differences of cortical thickness between mouse and rat [37, 63]. Finally, a simplified artificial capillary bed (Cs) consisting of a stacked hexagonal network with uniform diameters, lengths and tortuosity was added mimicking the typical highly interconnected and mesh-like topology [2, 3, 48, 64]. The values for capillary diameters (4 μm [3]), lengths (62 μm) and tortuosity (1.37 [3, 48, 65]) were chosen such that the overall length and volume densities of the vasculature are within the range observed *in vivo* (see S3 Table). By connecting the leaf vertices of the penetrating trees to the respective closest capillaries, we obtained a full network which represents the typical hierarchical structure of realistic microvascular networks, where blood sequentially flows through SAs, DAs, Cs and AVs [64]. As such, our approach enables fast and straightforward generation of large microvascular networks, which are based on realistic pial vascular topologies and represent the overall characteristics of the *in vivo* microvasculature. More details on the validation of our semi-realistic networks are presented at a later stage.

### Blood flow model

As commonly done in previous works by our group [37, 38, 42–44] and others [39, 49, 66–68], we represented the brain vasculature by a network consisting of edges and vertices, which correspond to individual blood vessels and connections of at least two blood vessels. Poiseuille’s law was used to compute blood flow rates, RBC velocities and pressures in the entire network. As our focus is not on local capillary perfusion characteristics [37] but on overall flow changes, we neglected the effects of phase-separation [69] and assumed a constant haematocrit of 0.3. The impact of haematocrit and vessel diameter on flow resistance is considered by the formulation of Pries et al. [70]. Please refer to S2 Appendix for a more detailed description of the blood flow model.

Pressure boundary conditions of 100 and 10 mmHg [37] were assigned to in- and outflow vertices, respectively, and kept constant for all simulations, i.e., at baseline, MCAo, MCAo & LMC-dil and MCAo & LMC/SA/DA-dil. The networks were constructed such that only one inflow vertex exists at the most upstream SA vessels at the circle of Willis (CoW in Fig 1C). Outflow boundary conditions were assigned to all AV tree root vertices [37].

### Tuning of vessel diameters to match experimental data

As described in the previous sections, the three-dimensional semi-realistic microvascular network (Fig 1D) mimics the topology and structure of the real vasculature and is consistent with case-specific pial networks from *in vivo* experiments. However, the uncertainties related to vessel diameters are still large. To reduce these uncertainties, additional data from *in vivo* measurements and literature were incorporated into the model.

**Fig 2:**
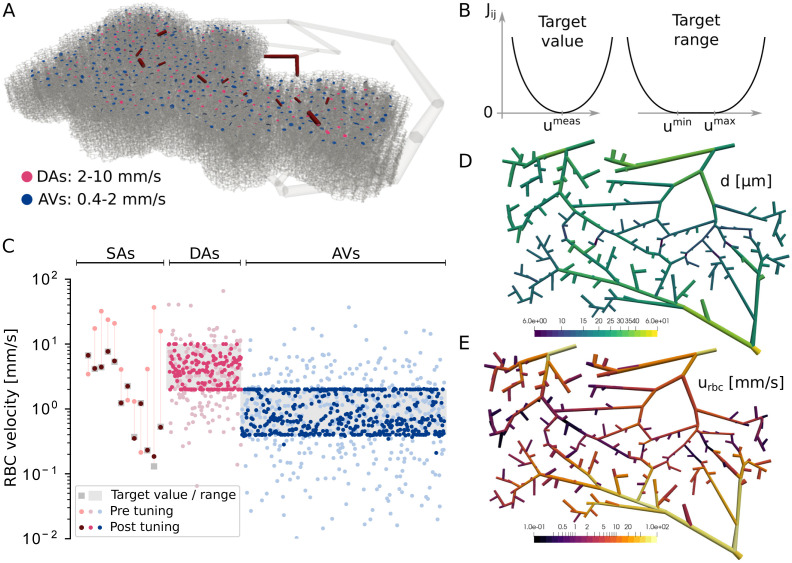
Inverse modelling approach. (A) Network C57BL/6_I_, where red edges represent SAs with *in vivo* RBC velocity measurements. Furthermore, purple and blue dots highlight the locations of DA and AV roots, where a range for the RBC velocities is prescribed. (B) Illustration of the two types of cost functions used in this work for prescribing target values (left) or ranges (right) for the RBC velocities. (C) RBC velocities in SAs, DAs and AVs before and after tuning. (D-E) Diameters (D) and RBC velocities (E) at the SAs after tuning the semi-realistic network (see also S11 Fig).

In previous work we presented an inverse modelling approach to estimate diameter responses necessary to locally upregulate blood flow during functional activation [43]. Here, we adapted this inverse model and tuned the diameters of the entire network by prescribing constraints on RBC velocities in selected blood vessels. We used two different types of velocity constraints: 1) Constraints that prescribe a precise target velocity $u_{ij}^{meas}$ or 2) a target range with a defined minimum $u_{ij}^{min}$ and maximum $u_{ij}^{max}$ velocity for edge *e*_*ij*_. Precise target velocities were used in SAs where two-photon line scan RBC velocity measurements from the *in vivo* experiments were available (Fig 1A, S1 Table). If no velocity measurement was available for a LMC, the target value was set to the median velocity measured across all other LMCs, i.e., $u_{ij}^{meas}=0.53\;\text{mm}/s$. Additionally, in agreement with *in vivo* data [27, 71], we prescribed a range of 2–10 mm/s for the RBC velocities in the most upstream DAs (DA roots) and a range of 0.4–2 mm/s in the AV vessels at the outflow boundaries (AV roots). A visualisation of all blood vessels with a prescribed target RBC velocity is given in Fig 2A.

The inverse problem was solved by iteratively minimizing a cost function describing the previously mentioned velocity constraints. Here, we used a sum of parabolic functions (Fig 2B) with cost function values zero at the precise target values $u_{ij}^{meas}$ or in the range from $u_{ij}^{min}$ to $u_{ij}^{max}$, respectively. Similar to our previous publications [43, 72], we searched for a minimum of the cost function by using a gradient-based approach, where the adjoint method was applied (see S3 Appendix for more details). The converged solution of the inverse problem is ambiguous since the parameter space is much larger than the number of constraints, i.e., diameters of 204’116 blood vessels can be adjusted to match 641 constraints in SAs, DAs and AVs of the network C57BL/6_I_ (Fig 2A). Therefore, an entire manifold of different combinations of vessel diameters solve the inverse problem, and thus infinite many solutions with the cost function value of zero exist [43, 72]. The validation of topology and flow field against literature data (see subsequent section) allows us to be confident that our semi-realistic microvascular networks are a good approximation of the cortical vasculature. Moreover, while the current cost function only contains constraints for RBC velocities in a subset of blood vessels, it could easily be extended by additional terms to further reduce the ambiguity of the solution space [43, 72, 73]. To account for the different levels of uncertainties related to the diameters of the initial network, the maximum diameter change compared to prior values was restricted and varied depending on vessel categories, i.e., ±5% for SAs with diameter measurements, ±20% for SAs without diameter measurements and ±50% for all other vessel types (DAs, Cs and AVs).

The comparison of diameter distributions before and after tuning (S11(A)–S11(C) Fig) shows that applying the inverse model mainly affected SAs. Changes of other vessel types were also observed, however, they were mainly located close to penetrating trees and in the upper layers of the networks (S11(D)–S11(F) Fig). Note that with our inverse modelling approach it is expected that the largest diameter changes are close to the measurements, because the cost function is minimised by adjusting the parameters with the highest sensitivity (S3 Appendix).

Fig 2C demonstrates that the velocities in SAs after tuning the vessel diameters agree well with the target values from the measurements. Furthermore, the velocities in DA and AV roots are now within the prescribed velocity ranges. This demonstrates that using an inverse model for optimizing the network is an effective tool to improve the overall quality and to obtain RBC velocity distributions that are consistent with given experimental data. The diameters and RBC velocities after tuning in SAs of C57BL/6_I_ are visualised in Fig 2D and 2E.

### Validation of semi-realistic microvascular networks

The final semi-realistic microvascular networks of all four datasets are visualised in Fig 1D–1G. On average, the networks span an area of 28.75 mm^2^ and consist of approximately 200’000 edges. A more detailed summary of network characteristics is available in S2 Table. We validated the networks by comparing different vessel and flow characteristics to literature values. Overall, the length and volume densities, as well as the mean diameters of SAs, DAs, Cs and AVs, are within physiological range in all four networks (S3 Table). The flow field in the vasculature is highly heterogeneous [37, 64, 74], which results in order of magnitude differences of RBC velocities in individual vessels. Generally, both the magnitudes (S4 and S28 Tables) and heterogeneity (S12 and S13 Figs) of velocities and RBC flow rates are captured well by our simulations and agree with experimental data from literature. Furthermore, our networks agree with reported values for cerebral blood flow (S27 Table) and wall shear stresses in different vessel types (S29 Table).

### Adjustment of vessel diameters to represent the effects of MCA occlusion, LMC dilation and artery dilation

To mimic a stroke at the level of the MCA (MCAo) we constricted an edge segment with a length of 50 μm upstream to the pial network to 10% of its baseline diameter (MCAo in Fig 1D–1G). This occlusion is at the MCA-M2 bifurcation [61] and induces at significant pressure drop. The semi-realistic microvascular networks described above are located further downstream in the M4/M5 area and of course are affected tremendously by the MCAo. The precise response of vessels in the M4/M5 area (both on the MCA and the neighboring ACA side) is still not fully understood and likely a mix of dilations and constrictions [17, 27, 28]. Further confounding factors are the precise time point after stroke and potential effects of anesthesia [75]. In any case, the alterations in diameter are likely a result of a multitude of response mechanisms, including some form of autoregulation, passive changes due to vessel elasticity and potentially additional pathological adaptations.

As our focus is on flow redistribution immediately after stroke and the effect of LMCs, we decided to limit vascular adaptations to three clearly defined states: 1) no diameter adaptations except MCAo, 2) MCAo and LMC dilation (MCAo & LMC-dil) and 3) MCAo & LMC-dil and SAs and DAs dilation (MCAo & LMC/SA/DA-dil). While state 1) & 2) represent the situation immediately after stroke, state 3) is intended to mimic simplified forms of either autoregulation or therapeutic intervention causing arteriole dilation by vasodilators [76, 77] or stimulation [22, 23].

The diameters of LMCs for the state MCAo & LMC-dil were derived from the *in vivo* experiments, when such data was available (C57BL/6_I_ in Fig 1A and S1 Table). For all other scenarios, a uniform dilation factor of 1.7 was assumed, which corresponds to the median dilation factor of the C57BL/6_I_ network. For the state MCAo & LMC/SA/DA-dil, SAs and DAs were dilated by 10%, which is the average peak dilation observed *in vivo* for pial vessels during functional hyperaemia [64]. Note that models for autoregulation are available [78, 79]. However, as autoregulation is likely impaired during stroke [80] we decided to work with a more general state that allows us to analyze how SA & DA dilations could counteract the flow and pressure reductions caused by the MCAo.

For all states we accounted for the elasticity of blood vessels by using a pressure-area relationship based on linear elastic theory [21, 79, 81] to estimate passive diameter changes due to pressure alterations in response to MCAo. Such pressure alterations are observed across large parts of the vasculature (S6(A) and S6(C) Fig). Interestingly, despite the large changes in pressure, the calculated constrictions due to vessel elasticity were generally below 10% (S4 Fig) and the elasticity of vessels only slightly affected the results compared to when all vessels were assumed rigid (see S21 Table, S22 and S23 Tables). For more details on the vessel elasticity model, please refer to S4 Appendix.

It is important to note that the defined post-stroke states are a strong simplification in comparison to the complex situation *in vivo*. However, at the same time, these clearly defined states offer the advantage that simulation results can be compared rigorously and that it is possible to comment on the isolated effects of specific alterations, e.g. LMC-dilation. This would be challenging for states including all post-stroke vasodynamics at once.

### Variation of LMC densities

One of our key goals was to quantify the impact of the number of LMCs on flow redistribution after MCAo, MCAo & LMC-dil and MCAo & LMC/SA/DA-dil. As the overall network structure differs between individual data sets, it is difficult to directly compare networks with and without LMCs. Consequently, we exploited the benefits of *in silico* investigations and modified the network characteristics by removing selected or all LMCs from C57BL/6 networks, and analogously adding any desired number of LMCs to BALB/c networks. This allows us to create additional scenarios with varying LMC densities for all four networks. Subsequently, we analysed how the presence of many (100% LMC), few (50% LMC) or no (0% LMC) LMCs affects the overall perfusion changes in an isolated manner, i.e., without any other differences between the networks. For the C57BL/6 networks, the 50% LMC and 0% LMC scenarios were defined by randomly removing half or all existing LMCs, respectively (S5(A) Fig). Accordingly, LMCs were added to the BALB/c networks based on the LMC characteristics derived from the two C57BL/6 datasets (see S5 Fig for details).

## Results

With our simulations we studied how the occlusion of the MCA (MCAo), the subsequent dilation of LMCs (MCAo & LMC-dil) and the additional dilation of all arteries (MCAo & LMC/SA/DA-dil) affect the distribution of blood flow compared to baseline (Base). We first focus on results obtained for networks characterised by many LMCs (C57BL/6_I_ and C57BL/6_II_) and analysed the changes in blood flow rates in SAs, DAs and Cs. The results are then compared to networks with fewer or no LMCs, which provides further insights on the role of LMCs during stroke. To facilitate comparison, the results for MCAo & LMC/SA/DA-dil are depicted together with the other states. However, to focus on the impact of arterial dilations, the description of this state is addressed in a separate section, i.e., after describing perfusion changes at the level of SAs, DAs and Cs in response to MCAo and LMC-dil.

### An occlusion of the MCA passively increases the flow rates in LMCs and ACA sided SAs

**Fig 3:**
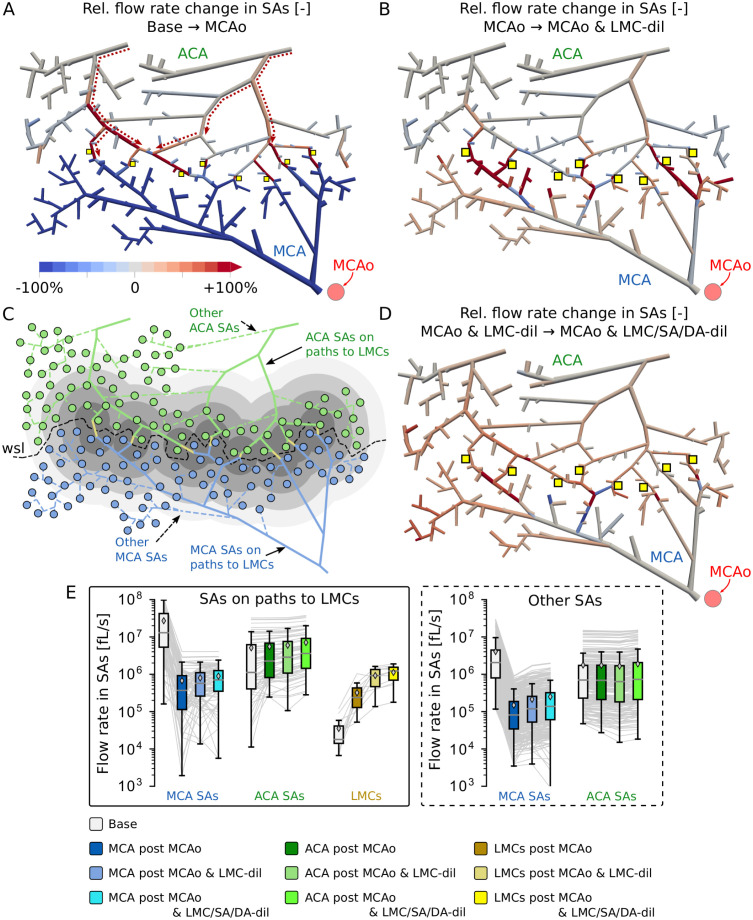
Flow rate changes in SAs in response to MCAo and subsequent LMC-dil and SA/DA-dil. (A-B) Relative changes of blood flow rates in individual SAs of the network C57BL/6_I_ after MCAo compared to baseline (A) and after LMC-dil in comparison to MCAo only (B). (C) Classification of SAs and DAs into MCA and ACA sided vessels. Additionally, the SAs are classified into vessels located on a *shortest path to LMCs* (solid lines) and *others* (dashed lines). The grey shaded patches group DAs into different categories according to their planar distances to LMCs (spacing between differently coloured patches is 250 μm). The wsl describes the interface between MCA and ACA territory, which was extracted based on the Voronoi tessellation around all DA roots (Fig A panel B in S1 Appendix). (D) Relative changes of blood flow rates in individual SAs of the network C57BL/6_I_ after dilating all arteries (MCAo & LMC/SA/DA-dil compared to MCAo & LMC-dil only). (E) Flow rates in individual SAs (grey lines) at baseline, MCAo, MCAo & LMC-dil and MCAo & LMC/SA/DA-dil for both C57BL/6 networks, according to their classification from panel (C) (*SAs on paths to LMCs* in left panel, *others* in right panel). Boxplots and mean values (◇-symbol) are shown for each category.

In Fig 3A the relative changes of blood flow rates after MCAo compared to baseline are visualised for the network C57BL/6_I_ (see S1(A) Fig for C57BL/6_II_). As expected, the flow in MCA sided SAs was reduced drastically due to the upstream stroke. The opposite trend was observed in ACA sided SAs, where the flow rate mostly increased. This was most apparent in vessels on a direct flow path from the ACA to the LMCs (doted arrows) and consequently, the flow in all LMCs also increased substantially. Interestingly, this happened without dilating any vessels and follows directly from the changed pressure field due to the MCAo. More precisely, the pressures at baseline were approximately similar in MCA and ACA sided SAs, which leads to low residual flow rates in the LMCs (Figs 2E and 3E left). In contrast, the overall pressure in all MCA sided SAs was reduced drastically due to the MCAo (change of mean pressure $\Delta p_{rel}^{Base\to MCAo}=-60.0\%$ for C57BL/6_I_) (see S5 Table and S6 Fig). However, while the pressure also dropped in all ACA sided SAs, the corresponding mean pressure reduction was less pronounced ($\Delta p_{rel}^{Base\to MCAo}=-6.9\%$ for C57BL/6_I_). The resulting pressure difference between MCA and ACA induced a flow in ACA SAs towards the LMCs. Additionally, the altered pressure field caused flow direction changes in all LMCs and several other SAs on both MCA and ACA side (S2 Fig). This resulted in a unidirectional flow over the LMCs from ACA to MCA side after MCAo and in a shift of the watershed line towards the MCA territory.

To further analyse the results of both C57BL/6 networks quantitatively we classified the MCA and ACA sided SAs into different categories (Fig 3C), i.e., into vessels located on a shortest path from the inflow (CoW) to the LMCs (*MCA/ACA SAs on paths to LMCs*) and into other vessels (*Other MCA/ACA SAs*). Fig 3E shows the flow rates for the networks with LMCs (C57BL/6_I_, C57BL/6_II_) for the four simulated scenarios (baseline, MCAo and MCAo & LMC-dil, MCAo & LMC/SA/DA-dil). The MCAo caused a drastic flow reduction in the majority (C57BL/6_I_: *n*_*rel*_ = 99.4%, C57BL/6_II_: *n*_*rel*_ = 100%) of MCA SAs, independently of whether they are located on a shortest path to LMCs or not. For example, in C57BL/6_I_ the relative changes of mean flow rate in *MCA SAs on paths to LMCs* and in *Other MCA SAs* were $\Delta q_{rel}^{Base\to MCAo}=-95.5\%$ and $\Delta q_{rel}^{Base\to MCAo}=-94.0\%$, respectively. Interestingly, different trends were observed for ACA sided SAs, where the mean flow rate in vessels located on *paths to LMCs* increased after MCAo ($\Delta q_{rel}^{Base\to MCAo}=+15.0\%$ for C57BL/6_I_) and slightly decreased in *others* ($\Delta q_{rel}^{Base\to MCAo}=-2.7\%$). Even though the average flow rate decreased in ACA SAs not on a path to LMCs, there are a few vessels where we also saw an increase in flow (C57BL/6_I_: *n*_*rel*_ = 11.0% of *Other ACA SAs*, C57BL/6_II_: *n*_*rel*_ = 28.6%). S5 and S6 Tables provide a detailed summary of results in C57BL/6_I_ and C57BL/6_II_.

In LMCs, the relative increase in mean flow rate due to MCAo was tremendous ($\Delta q_{rel}^{Base\to MCAo}=+758.5\%$ for C57BL/6_I_) (Fig 3E left and S6 Table). However, these large values were not only caused by the substantial increase in absolute flow rates, but also by the small baseline values in LMCs. Nevertheless, the results clearly demonstrate that a passive redistribution of flow at pial level occurs after MCAo without additional diameter adaptations.

The effect of dilating the LMCs in addition to MCAo is visualised in Fig 3B for C57BL/6_I_ (see S1(B) Fig for C57BL/6_II_). As expected, the dilation further increased the flow in all LMCs compared to MCAo only ($\Delta q_{rel}^{MCAo\to MCAo\&LMC-dil}=+91.0\%$ for C57BL/6_I_). This resulted in partial flow recovery in the majority of MCA sided SAs ($\Delta q_{rel}^{MCAo\to MCAo\&LMC-dil}=+22.3\%$ for C57BL/6_I_). However, please note that also after LMC-dil the flow rates were still substantially lower than at baseline ($\Delta q_{rel}^{Base\to MCAo\&LMC-dil}=-93.9\%$). The vessels close to the LMCs benefited predominantly from the redistribution of flow from ACA to MCA sided SAs. Moreover, LMC-dil generally improved the perfusion of both *MCA SAs on paths to LMCs* and *Other MCA SAs* (Fig 3E). This was because dilating the LMCs increased the pressure level in MCA sided SAs compared to MCAo ($\Delta p_{rel}^{MCAo\to MCAo\&LMC-dil}=+3.5\%$ in C57BL/6_I_, see S5 Table and S6 Fig). The resulting larger pressure difference from MCA sided SAs towards the capillary bed was the driving factor for the observed increase in flow in MCA SAs that did not benefit from a flow redistribution over the LMCs.

Dilating the LMCs subsequently to MCAo also increased the mean flow rates in *ACA SAs on paths to LMCs* (Fig 3E left). This is consistent with the improved perfusion of LMCs, which after stroke were exclusively supplied by ACA sided vessels. In *Other ACA SAs* not on a path to LMCs, the dilation of LMCs caused a reduction in flow (Fig 3E right), originating from the lower pressure level in ACA SAs (S5 Table and S6 Fig) and the resulting smaller pressure difference towards the capillary bed. Relative changes for C57BL/6_II_ are available in S5 and S6 Tables.

While the overall trends were comparable for both networks with LMCs (S6 Table), the precise value of the relative changes is affected by the network topology. Key factors for the resulting relative flow changes in response to MCAo and LMC-dil are the number of LMCs (S2 Table), the vessel connectivity and the size of the MCA and ACA region used in the *in silico* study (S2 Table).

### LMC dilation after MCAo improves flow in MCA DAs and Cs, but causes a further reduction in ACA DAs and Cs

**Fig 4:**
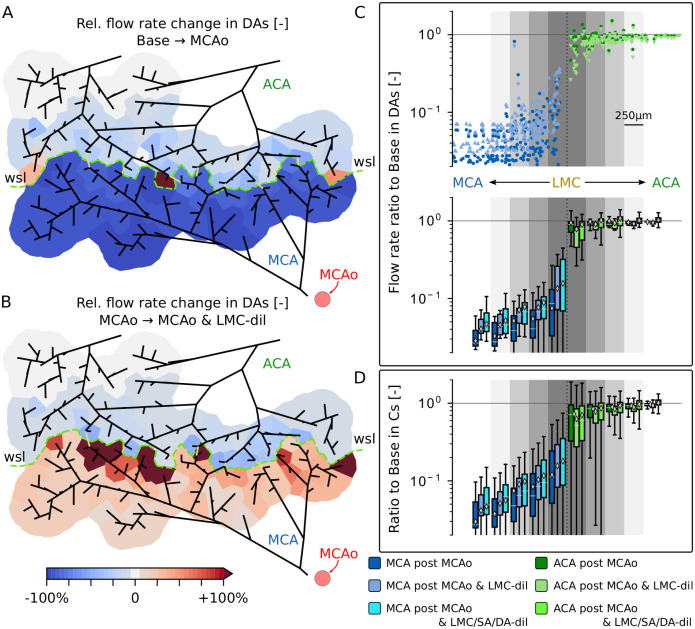
Flow rate changes in DAs and Cs in response to MCAo and subsequent LMC-dil and SA/DA-dil. (A-B) Relative perfusion changes in DA trees of the network C57BL/6_I_ after MCAo compared to baseline (A) and after MCAo & LMC-dil in comparison to MCAo only (B). The feeding area of each DA tree is approximated by the Voronoi polygons given by the tessellation around all DA roots (consistent with Fig A panel B in S1 Appendix). Each polygon is colour-coded based on the flow rate changes through the corresponding DA roots. Refer to S8(A) Fig for results after SA/DA-dil. (C) Top: Scatter plot with flow rate ratios “MCAo to baseline” (∘, dark colours) and “MCAo & LMC-dil to baseline” (△/▽, light colours) for the DAs of both C57BL/6 networks. △ and ▽ symbols are used to indicate whether the flow rate is increased or reduced after dilating the LMCs in comparison to MCAo only. The grey shading indicates the bins used to categorise the data points with respect to their distances to the LMCs according to Fig 3C. Bottom: Boxplots and mean values (◇-symbol) summarising the flow rate ratios for the different distance categories. Note that the flow rate ratios “MCAo & LMC/SA/DA-dil to baseline” are also shown here. (D) Flow rate ratios “MCAo to baseline”, “MCAo & LMC-dil to baseline” and “MCAo & LMC/SA/DA-dil to baseline” for Cs of both C57BL/6 networks.

The results above illustrate how flow was redistributed in SAs after stroke. However, the redistribution at the pial level is not directly equivalent to perfusion changes in penetrating vessels and the capillary bed, which are the key locations for oxygen and nutrient supply to the tissue. Consequently, we will now focus on the effects of MCAo and LMC-dil in DAs and Cs. Fig 4A visualises the relative change in flow in DA trees after MCAo compared to baseline for the network C57BL/6_I_ (results for C57BL/6_II_ in S3 Fig). Since the blood flow through the DA roots is conserved and eventually reaches the Cs, Fig 4A also provides an estimate of local perfusion changes in the capillary bed. The results show a reduced blood flow in almost all DAs after MCAo. However, the level of reduction is different on the two sides of the watershed line. While the integral flow rate through all MCA DAs was reduced by $\Delta Q_{rel}^{Base\to MCAo}=-92.6\%$ (C57BL/6_I_), the reduction in ACA DAs was only $\Delta Q_{rel}^{Base\to MCAo}=-5.7\%$. Importantly, the observed passive flow increase in ACA sided SAs close to LMCs (Fig 3A) did not translate to a flow increase in the underlying DAs. In contrast, the flow generally decreased in most DAs except in a few trees close to the watershed line. This is a first example, where the changes at the level of SAs differ from what is observed in DAs and therewith underlines the necessity to analyse perfusion changes for different vessel types separately.

The effect of additionally dilating the LMCs after MCAo is visualised in Fig 4B. In line with the results for MCA sided SAs (Fig 3B), a partial recovery of flow was observed in MCA DAs due to the improved flow redistribution over the LMCs and the changed pressure characteristics after the dilation (S5 Table and S6 Fig). This resulted in an increase in integral flow in MCA sided DAs by $\Delta Q_{rel}^{MCAo\to MCAo\&LMC-dil}=+38.9\%$ (C57BL/6_I_), which however was still substantially lower than at baseline. In contrast, in ACA sided DAs the dilation caused a further overall flow reduction of $\Delta Q_{rel}^{MCAo\to MCAo\&LMC-dil}=-5.0\%$ (C57BL/6_I_). Refer to S7 Table for a summary of integral results in DAs of both C57BL/6_I_ and C57BL/6_II_.

To better analyse how flow rates change locally, the flow rate ratios “MCAo to baseline” and “MCAo & LMC-dil to baseline” are shown in Fig 4C for individual DAs of both C57BL/6 networks as a function of distance to the LMCs. Generally, the reduction in flow in MCA sided DAs was more pronounced further away from LMCs and LMC-dil had a larger impact on the recovery of flow in MCA sided DAs close to LMCs. Therewith, the integral flow increase compared to MCAo was $\Delta Q_{rel}^{MCAo\to MCAo\&LMC-dil}=+63.9\%$ (C57BL/6_I_) if only vessels within a maximum distance of 250 μm to the LMCs were considered. The opposite trend was observed for ACA sided DAs close to LMCs, where the flow reduction due to LMC-dil is more severe within 250 μm to LMCs ($\Delta Q_{rel}^{MCAo\to MCAo\&LMC-dil}=-33.4\%$). In addition to differences between vessel types, these results clearly show that even within one vessel type perfusion changes vary in function of the exact topological location of the DA.

The overall trends for capillaries (Fig 4D and S8 Table) were generally comparable to the results in DAs. Nonetheless, the integral flow reduction after MCAo was slightly less pronounced in MCA sided Cs and slightly larger in ACA Cs in comparison to MCA and ACA sided DAs, respectively. This resulted in a smoother transition between the integral perfusion drops in MCA and ACA sided Cs close to LMCs, which is plausible because capillaries in the watershed area can be fed simultaneously by MCA and ACA DAs. Importantly, the response at the level of individual capillaries is very heterogeneous and flow increases, decreases and also reversals have been observed. This provides evidence for a significant redistribution of blood flow within the capillary bed. Moreover, the reduced drop in integral perfusion for MCA sided Cs close to the wsl showed that the high interconnectivity of the capillary bed offers some robustness during MCAo, which would be difficult to capture if flow changes were only analysed in DAs or individual Cs.

In summary, the partial recovery of flow in MCA sided DAs and Cs can be augmented by dilating LMCs. However, this comes at the cost of a reduced perfusion of ACA sided DAs and Cs, which likely however is not so severe that under-perfusion is to be expected for ACA sided vessels.

### Dilation of arteries after stroke improves the perfusion of MCA and ACA sided vessels

The dilation of all arteries subsequent to MCAo & LMC-dil improved the perfusion of the majority of MCA and ACA sided SAs (Fig 3D and S7(A) Fig). More precisely, while only dilating LMCs after MCAo resulted in an increase in flow by $\Delta q_{rel}^{MCAo\to MCAo\&LMC-dil}=+22.3\%$ in all MCA SAs and $\Delta q_{rel}^{MCAo\to MCAo\&LMC-dil}=+1.2\%$ in all ACA SAs of the network C57BL/6_I_ (S6 Table), the dilation of SAs and DAs further augmented the flow recovery to $\Delta q_{rel}^{MCAo\to MCAo\&LMC/SA/DA-dil}=+46.2\%$ (MCA SAs) and $\Delta q_{rel}^{MCAo\to MCAo\&LMC/SA/DA-dil}=+16.8\%$ (ACA SAs), respectively (S14 Table). Moreover, an increase in mean flow rates after SA/DA-dil was consistently observed in *MCA/ACA SAs on paths to LMCs*, *Other MCA/ACA SAs* and LMCs (Fig 3E). This goes hand in hand with a higher pressure in SAs close to LMCs (S7(B) and S7(C) Fig and S13 Table).

The higher flow rates in SAs after SA/DA-dil directly translated into higher integral flow rates through DAs and Cs in both MCA and ACA territory (Fig 4C and 4D and S15 and S16 Tables). However, the local variability was high and flow increases as well as decreases were observed throughout the network (S8 Fig), which can be explained by local pressure changes in SAs and Cs (S7(B) and S7(C) Fig). This resulted in a partial compensation of the flow rate reductions in ACA sided vessels, which we observed after only dilating LMCs (Fig 4B). Thus, while LMC-dil reduced the overall flow rate in ACA Cs by $\Delta Q_{rel}^{MCAo\to MCAo\&LMC-dil}=-5.2\%$ compared to MCAo (S8 Table), additionally dilating SAs and DAs increased the mean perfusion by $\Delta Q_{rel}^{MCAo\to MCAo\&LMC/SA/DA-dil}=+2.8\%$ (S16 Table).

Taken together, dilating arteries subsequent to stroke improved the perfusion of all vessel types and across the entire vasculature. This can be expected since larger vessel diameters automatically reduce the flow resistance, and thus affect pressures and flow rates throughout the network.

### The impact of having many, few or no LMCs

**Fig 5:**
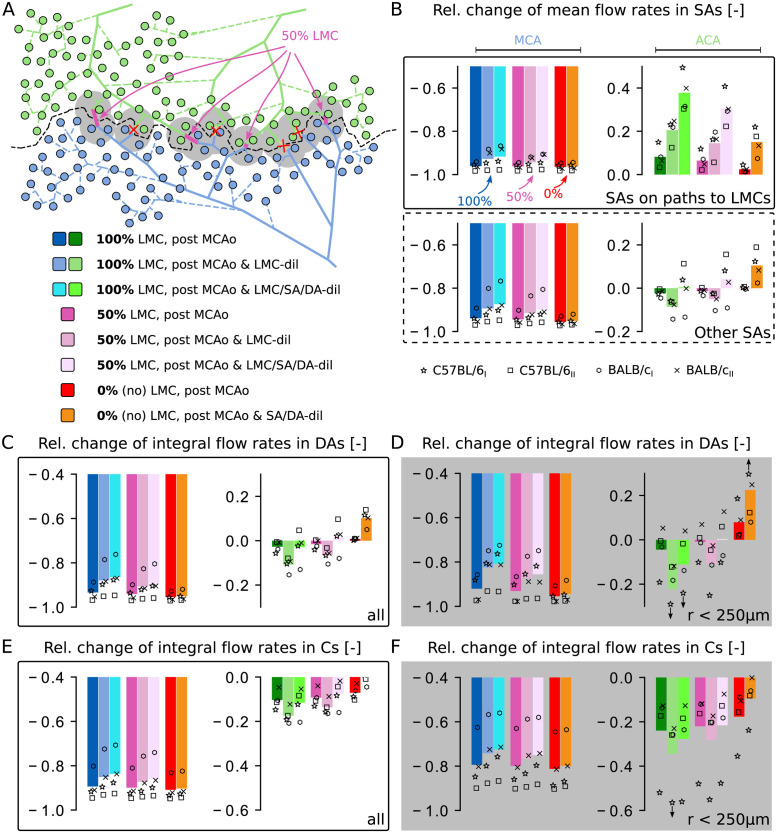
Impact of the number of LMCs on the overall flow rate changes in different vessel types. (A) Top: Map of the network C57BL/6_I_ and visualisation of the three scenarios with many, few and no LMCs. For the 50% scenario, only the pink-coloured LMCs are kept. The grey shaded area highlights the region within 250 μm to LMCs. Bottom: Colour map for the results displayed in panels B-F. (B) Relative changes of mean flow rates in MCA and ACA SAs of individual networks (markers) and averaged over all datasets (bars). The SAs are classified into vessels on a *shortest path to LMCs* (top) and *others* (bottom) (see Fig 3C). (C-D) Relative changes of integral flow rates computed over all MCA and ACA sided DAs (C) and for DAs located within 250 μm of LMCs only (D). The integral flow rate is defined as the sum of flow rates over all vessels of the respective vessel category (S7 Table). (E-F) Relative changes of integral flow rates in Cs in the entire network (E) and within 250 μm of LMCs (F).

The results presented above (Figs 3 and 4) focused on blood flow distributions after stroke in networks with LMCs. In the following, we wanted to understand the influence of LMCs on flow redistribution, and how flow characteristics changed in networks with few or without LMCs. To attain this goal, we vary the amount of LMCs in each of the four datasets to obtain three networks per dataset that are characterised by many, few or no LMCs (see Fig 5A for C57BL/6_I_ and S5(A)–S5(C) Fig for C57BL/6_II_, BALB/c_I_ and BALB/c_II_). Since the number of LMCs is the only difference between the three networks of each dataset, the comparison of results provides direct insights on the role of LMCs during stroke.

#### Pial arteries

The impact of the number of LMCs on mean flow rate changes during stroke in *MCA/ACA SAs on paths to LMCs* and *Other MCA/ACA SAs* is shown in Fig 5B. For *MCA SAs on paths to LMCs* (Fig 5B top) the differences for the average reduction in flow after MCAo were small for the tested scenarios (S9 Table). However, more LMCs lead to a larger average flow increase after LMC-dil, i.e., $\langle \Delta q_{rel}^{MCAo\to MCAo\&LMC-dil}\rangle =+78.8\%$ for 100% LMC and $\langle \Delta q_{rel}^{MCAo\to MCAo\&LMC-dil}\rangle =+51.9\%$ for 50% LMC compared to MCAo (average values computed over all datasets). The same trends were also observed after SA/DA-dil, where the average flow rate increased by $\langle \Delta q_{rel}^{MCAo\to MCAo\&LMC/SA/DA-dil}\rangle =+105.1\%$ for 100% LMC and $\langle \Delta q_{rel}^{MCAo\to MCAo\&LMC/SA/DA-dil}\rangle =+77.0\%$ for 50% LMC compared to MCAo (S18 Table). Larger differences were observed for *ACA SAs on paths to LMCs*, where the increase in flow after MCAo was more pronounced in networks with many LMCs ($\langle \Delta q_{rel}^{Base\to MCAo}\rangle =+8.2\%$ for 100% LMC, +6.5% with 50% LMC and +2.4% with 0% LMC). In line with these results, LMC-dil and SA/DA-dil led to a higher flow rate increase in networks with more LMCs (S9 and S18 Tables). This is because both more LMCs and dilating vessels reduce the overall resistance of parallel arranged vessels, which directly translates into higher flow rates. Interestingly, we also observed a small flow increase after MCAo in *ACA SAs on paths to LMCs* in networks with no LMCs (0% LMC). This can be explained by the reduced pressure in ACA sided Cs close to the wsl.

The pressure changes in SAs after MCAo, and the resulting pressure differences towards the capillaries, explain the impact of LMCs on *Other MCA/ACA SAs* (Fig 5B bottom): The average reduction in mean pressure in MCA SAs due to MCAo was more severe with fewer LMCs (S10 Table). This resulted in lower flow rates in *Other MCA SAs* for approximately similar pressures in the capillary bed (S9 Table). The opposite trend was observed for *Other ACA SAs* after MCAo, where pressures (S10 Table) and flow rates (S9 Table) decreased more in networks with many LMCs. These pressure changes were amplified by dilating LMCs, which translated to larger flow changes in *Other ACA SAs* after MCAo & LMC-dil compared to MCAo. After dilating DAs and SAs, the pressure level slightly increased for most scenarios (S17 Table) compared to MCAo & LMC-dil (S10 Table). This generally improved the mean flow rates in both MCA and ACA sided SAs. While this trend is consistent for all numbers of LMCs (S18 Table), larger differences were observed for the cases with more LMCs.

In summary, a higher number of LMCs increased flow redistribution from the ACA to the MCA side. This is most apparent from the increased flow rates in ACA SAs on paths to LMCs and the flow reductions in other ACA SAs. These trends are comparable across the four datasets, even though the variability with respect to mean flow rate changes is relatively large. As previously stated, this is caused by topological differences across the four datasets, e.g. the number of MCA and ACA sided SAs, the number of LMCs and the DA tree density.

#### Descending arteries and capillaries

Fig 5C shows the relative change of integral blood flow rates through all MCA and ACA sided DAs (see S11 and S19 Tables for a summary of averaged results). The differences between networks with different number of LMCs were qualitatively consistent with the results for *Other MCA/ACA SAs*. This is expected, since downstream of SAs blood eventually flows into DA trees and capillaries. A closer look at DAs within a planar distance of 250 μm to LMCs (Fig 5D) revealed that flow was reduced less in MCA DAs close to LMCs, especially in networks with 100% LMC or 50% LMC. The opposite behaviour was observed in ACA DAs, where flow dropped more close to LMCs and for a higher number of LMCs. This further confirms that LMCs redistribute blood from ACA DAs towards MCA DAs.

In line with the results in ACA sided SAs, we observed an increase in integral flow in ACA DAs close to LMCs for all four networks with 0% LMC (Fig 5D). As previously mentioned, this is caused by the reduced pressure in ACA sided Cs close to the wsl. Occasionally, this was also observed in networks with LMCs, predominantly in ACA DAs close to the watershed line connected to SAs that do not lead to LMCs (Fig 4A and S3(A) Fig).

Interestingly, we observed that dilating LMCs after MCAo has a larger effect on recovering flow in MCA sided DAs than dilating all arteries by 10%. For example, with 100% LMC, the observed flow rate increase of $\langle \Delta Q_{rel}^{MCAo\to MCAo\&LMC-dil}\rangle =+82.9\%$ (S11 Table) through all MCA sided DAs after LMC-dil was only increased by an additional 22.7% when also SAs and DAs where dilated, i.e., to $\langle \Delta Q_{rel}^{MCAo\to MCAo\&LMC/SA/DA-dil}\rangle =+105.6\%$ (S19 Table). More strikingly, the flow reductions in MCA DAs of networks with LMCs and only MCAo (no additional dilations, S11 Table), were less severe than for the case with 0% LMC but with SA/DA-dil (S19 Table). Furthermore, SA/DA-dil generally had a larger effect on recovering flow in the MCA territory as function of the number of LMCs. In contrast, in ACA sided DAs flow rates increased more in networks without LMCs after SA/DA-dil, because here no blood could be rerouted over collateral paths.

The results for DAs can be directly translated to Cs, as seen in Fig 5E for the entire network and in Fig 5F for vessels within 250 μm of LMCs (see S12 and S20 Tables for averaged results). One major difference to DAs is that the integral flow rates in Cs decreased after MCAo for all networks and for all numbers of LMCs. This was the case for both MCA and ACA sided Cs, independently of whether all (Fig 5E) or only vessels close to the watershed line (Fig 5F) were analysed. Thus, importantly, the flow increase in some DAs close to the watershed line did not translate to an overall higher integral capillary perfusion, but likely only affected the capillaries proximal to the respective DAs. Even though the MCAo had a much larger effect on the flow reduction in MCA Cs compared to ACA Cs, the differences were smaller than in DAs. This is because the entire capillary bed is highly interconnected and there is no sharp boundary between capillaries that are only fed by MCA or ACA, respectively. Consequently, individual capillaries may receive flow from multiple DAs, located at the MCA, ACA or both sides. This leads to a smoother transition between observations for MCA and ACA Cs, if compared to DAs.

As for SAs, the precise level of flow rate change in DAs and Cs varied across datasets due to differences in topology. Nonetheless, we consistently observed that more LMCs and LMC dilation reduce the drop in perfusion in MCA DAs and Cs during stroke. For MCA sided capillaries the integral perfusion increased by +57.2% in C57BL/6_I_, +32.5% in C57BL/6_II_, +63.5% in BALB/c_I_ and +88.0% in BALB/c_II_, if we compare the setup with 100% LMC and dilated LMCs to the case without LMCs. This goes hand in hand with a larger reduction in flow in ACA DAs and Cs for the case with many dilated LMCs (-10.9% in C57BL/6_I_, -7.9% in C57BL/6_II_, -15.5% in BALB/c_I_ and -9.6% in BALB/c_II_). Furthermore, dilating all arteries by 10% improved the perfusion of MCA sided DAs and Cs, and partially compensated the flow reductions in ACA sided vessels observed after LMC-dil. Taken together our results suggest that more LMCs and the dilation of LMCs is beneficial to maintain some perfusion during MCAo, especially close to the watershed line. While this redistribution of flow comes at the cost of a reduced perfusion on the ACA side, this drop is relatively small and likely does not cause any immediate tissue damage. Additional dilations of SAs and DAs help to further improve perfusion but rely on LMCs to distribute blood flow to the MCA side.

## Discussion

By performing blood flow simulations in large semi-realistic microvascular networks, we generate novel insights into the redistribution of blood flow in response to stroke. In networks with LMCs, we observed a pronounced increase in blood flow in all LMCs and a directed flow from ACA towards MCA sided SAs. It is noteworthy that these responses occurred without dilating any LMCs and directly resulted from the changed pressured field due to the occlusion of the MCA. Interestingly, the observed increase in flow in all LMCs and some ACA sided SAs after MCAo did not translate into a rise in integral perfusion of DAs and Cs, but to a redistribution of flow from ACA sided DAs to MCA sided DAs. This effect was most pronounced in networks with a large number of LMCs and was further enhanced by LMC dilation. It is also consistent with the experimentally [12] and numerically [21] observed smaller infarct volumes in networks with LMCs.

Additional dilation of all arteries after stroke increased flow rates in the entire vasculature, which is consistent with experimental studies showing that functional stimulation can protect the cortex after MCAo, likely by more efficiently re-routing flow via collateral vessels [22, 23]. We also observed that the effect of artery dilation on recovering flow in MCA sided vessels is more pronounced in networks with many LMCs. Even more strikingly, our simulations suggest that the sole existence of LMCs has a larger effect on reducing the perfusion drop in MCA sided vessels than dilating arteries. This suggests that therapeutic vasodilations have potential to further increase perfusion after stroke, but therapeutic success likely strongly depends on the extent of LMCs. This aspect is also a probable explanation why clinical studies have been reporting different results [76, 77].

Within our current set-up it is challenging to precisely asses to which extent LMC-dil and SA/DA-dil offer protection for the neural tissue in the stroke area. Even for MCAo & LMC/SA/DA-dil, perfusion in the capillary bed on the MCA side drops on average by -83.7%. While this is less than for MCAo in networks without LMCs (-90.9%), it still might lead to an energetic undersupply of neurons in the MCA area. A key factor to reduce tissue damage is certainly the time point at which perfusion can be re-established within the infarct area. In this regard, maintaining minimal perfusion to the infarct area might have the advantage that it could help to reduce secondary pathological alterations, such as capillary constrictions [82–84] or capillary stalls by neutrophils [15]. However, further *in vivo* and *in silico* studies will be necessary to quantify the protective capacity of LMCs and arterial dilations.

Another interesting observation is that the perfusion change in individual vessels in response to MCA occlusion, LMC dilation and artery dilation was highly heterogeneous. Differences were not only observed with respect to vessels types, but also with respect to the precise location of the vessel, e.g. if the SA is on a path to the LMCs or not. More precisely, at pial level an increase in perfusion predominantly occurred in ACA sided SAs located on a direct flow path from the ACA towards the LMCs. The flow rates in other ACA sided SAs remained approximately constant or decreased substantially in MCA sided SAs. At the level of MCA sided DAs and Cs we observed that vessels close to LMCs were generally affected less by the drop in perfusion than vessels further away. This is also interesting considering that recent work of our group revealed that reperfusion dynamics vary for the MCA-M3 and the MCA-M4/M5 territory [61]. While the precise origins of these differences are not yet fully understood, our current work already shows (on a even more local scale) that analysing flow changes at the level of SAs or in single DAs and Cs is not sufficient to obtain a complete picture of the perfusion change in the entire vasculature. This also highlights the necessity to account for the precise location of the vessel while interpreting both *in vivo* and *in silico* results and for the comparison of both. In this context, *in silico* approaches offer the advantage that flow changes can be analysed across all vessels and are not limited to a subset of vessels, as for example in two-photon microscopy.

Generally our observations are in agreement with experimental studies analysing perfusion changes during stroke in single LMCs [10, 13, 22, 28, 85, 86] and in SAs, where flow reductions, reversals or even increases have been observed [26, 27]. Also at the level of DAs [27, 28, 87] and Cs [87] a reduction in blood flow after stroke has been reported in *in vivo* experiments. Nonetheless, two important aspects have to be kept in mind if comparing our *in silico* data to *in vivo* experiments.

First, in the current study we only modelled how vessel diameters change passively after stroke, and additionally considered scenarios where LMCs and arteries were dilated to specified values. While this is an advantage to study the isolated effect of MCAo and LMCs and gives an impression on how perfusion changes immediately after stroke, it is also a strong simplification, because *in vivo* both active and passive constrictions and dilations are observed across SAs and DAs in response to stroke [15, 26–28]. Furthermore, at the level of capillaries, constrictions [17, 82–84] and blockages by neutrophils have been reported [15]. While it is evident that such alterations play a significant role for the resulting flow rate changes, their precise impact cannot be quantified until an in-depth description of all vasodynamics in response to stroke becomes available. For example, numerical models for autoregulation are available in literature. However, as autoregulation is impaired during stroke [80], it is currently unknown how these models would need to be adjusted in a post-stroke state. Nonetheless, incorporating such data into the presented *in silico* model is generally possible and would allow more refined studies on the redistribution of flow.

A second aspect that should be mentioned is that we focused on the analysis of relative flow rate changes. In *in vivo* experiments the measured quantity commonly is RBC velocity. For constant vessel diameters, the change in flow rate is identical to the change in velocity. However, if the vessel diameter is not constant the relative flow rate and RBC velocity change may differ. For example, in a dilated vessel the flow rate might increase, while the RBC velocities decreases. This has to be considered if comparing RBC velocity measurements against flow rate changes.

In the current study only few RBC velocity measurements and literature data were used. However, generally the inverse modelling approach allows to incorporate large numbers of *in vivo* measurements, e.g. from high-resolution tomographic imaging methods [32–35]. Moreover, the tuning of networks is not limited to velocity measurements, and other flow characteristics such as blood pressures, wall shear stresses or average flow rates measured on a coarser scale [43] could be added for tuning. This would further strengthen the link between *in vivo* experiment and *in silico* setup, and solidify the joint interpretation of results.

The presented simulation framework is not only versatile with respect to the type of incorporated measurements, but could also be employed to reduce uncertainties in fully realistic microvascular networks. For example, our inverse modelling approach could be used to improve diameter estimates in whole brain vascular reconstructions [45–48] based on reported structural and functional characteristics of the microvasculature, e.g. to make the networks consistent with observed flow rate distributions or vascular densities. In this context it is important to note, that inferring diameters of large networks based on limited experimental data inevitably results in ambiguous solutions, since multiple values of vessel diameters can match the prescribed data [43, 72, 73]. In the current study, we used a maximum allowable diameter change to reduce the ambiguity and validated the resulting networks with literature data. Alternatively, regularisation constraints could be applied to further reduce the space of possible solutions [72].

Besides addressing static scenarios, our approach could be extended to model reperfusion dynamics after clot removal. Possible applications would be to improve therapeutic interventions or strategies for clot removal and for avoiding reperfusion injuries [7, 16]. In addition to the aforementioned in-depth characterisation of the vasodynamics after MCAo, such studies would require a model to describe the dissolution of the clot. Based on our current observations the dilation of LMCs, and possibly also other SAs, has the potential to increase perfusion in the territory affected by MCA occlusion. Thus, vasodilations at the level of SAs could be a therapeutic target point to reduce the size of the under-perfused territory. Nonetheless, it has to be kept in mind that the area of impact is likely limited to regions close to the watershed line and that the flow increase on the MCA side paritally comes at the cost of reduced blood supply on the ACA side. This also raises the interesting question if for an increasing number of LMCs we would observe a saturation effect regarding the flow redistribution or if the overall stroke outcome due to local reductions in blood supply would worsen.

Taken together, to the best of our knowledge, we present the first simulation study in large semi-realistic microvascular networks derived from case-specific pial networks and consistent with *in vivo* velocity measurements. This strong link between *in silico* and *in vivo* data is a key benefit of our novel simulation framework that certainly will also be beneficial for other study designs. Moreover, our *in silico* approach offers the advantage that networks can be altered systematically and resulting perfusion characteristics can be analysed quantitatively. This is crucial considering that the variability of the vascular topology is large across different mouse strains and even in individual animals [11, 12, 18]. As such, our *in silico* approach allows more robust conclusions on the role of specific microvascular characteristics, as for example the number of LMCs. Because of the strong connection to case-specific *in vivo* data it is ideally suited to be employed hand-in-hand with *in vivo* experiments and to amplify the amount of conclusion to be drawn.

## Supporting information

S1 Fig Relative changes of blood flow rates in SAs. Relative changes of blood flow rates in individual SAs of the network C57BL/6_II_ from Base to MCAo (A) and MCAo to MCAo & LMC-dil (B). The yellow squares indicate the locations of the LMCs. Supplement to Fig 3A and 3B. Refer to S7(A) Fig for results after LMC/SA/DA-dil.
(PDF)

S2 Fig Direction changes in response to MCAo. Edges with direction changes (dark blue) in response to MCAo for the networks C57BL/6_I_ (A) and C57BL/6_II_ (B). The yellow squares indicate the locations of the LMCs. Supplement to Fig 3.
(PDF)

S3 Fig Relative changes of blood flow rates in DAs. Relative changes of blood flow rates in DAs of the network C57BL/6_II_ from Base to MCAo (A) and MCAo to MCAo & LMC-dil (B). Supplement to Fig 4A and 4B. Refer to S8 Fig for results after LMC/SA/DA-dil.
(PDF)

S4 Fig Relative changes of SA diameters in response to MCAo. Relative changes of SA diameters in response to MCAo in the networks C57BL/6_I_ (A) and C57BL/6_II_ (B).
(PDF)

S5 Fig Maps of pial networks and visualisation of the three scenarios with many, few and no LMCs. Maps of the pial networks C57BL/6_II_ (A), BALB/c_I_ (B) and BALB/c_II_ (C), and visualisation of the three scenarios with many, few and no LMCs. Supplement to Fig 5A. To define the number of added LMCs for the 100% LMC scenarios of the BALB/c datasets, the LMC density along the watershed line was computed from the two C57BL/6 datasets. The goal was then to obtain the same overall LMC density along the watershed line for the BALB/c networks. This was done with a sequential procedure by randomly selecting MCA DA vertices at the watershed line and connecting them to the closest DA vertices in the ACA territory (D). The sampled LMCs were then accepted or rejected based on two criteria derived from the C57BL/6 datasets: 1) The distance to already existing LMCs Δ_*c*_ was larger than 310 μm and 2) the maximum LMC length *l*_*c*_ was 1000 μm (E). As for the C57BL/6 networks, the 50% LMC scenario for BALB/c networks was defined by randomly removing LMCs.
(PDF)

S6 Fig Relative changes of pressures in SAs in response to MCAo and LMC-dil. Relative changes of pressures in SAs of the network C57BL/6_I_ from Base to MCAo (A) and MCAo to MCAo & LMC-dil (B). The results for the network C57BL/6_II_ are shown in panels (C) and (D), respectively. Refer to S7(B) and S7(C) Fig for results after LMC/SA/DA-dil.
(PDF)

S7 Fig Relative changes of flow rates and pressures in SAs in response to LMC/SA/DA-dil. (A) Relative changes of flow rates in SAs of the network C57BL/6_II_ from MCAo & LMC-dil to MCAo & LMC/SA/DA-dil. (B-C) The corresponding pressure changes are shown in panels (B) and (C) for both networks C57BL/6_I_ and C57BL/6_II_, respectively.
(PDF)

S8 Fig Relative changes of blood flow rates in DAs after LMC/SA/DA-dil. Relative changes of blood flow rates in DAs from MCAo & LMC-dil to MCAo & LMC/SA/DA-dil in the networks C57BL/6_I_ (A) and C57BL/6_II_ (B).
(PDF)

S9 Fig Reconstructions of surface artery networks. Experimentally acquired reconstructions of the surface artery (SA) networks BALB/c_I_ (A), BALB/c_II_ (B) and C57BL/6_II_ (C). The numbers refer to diameter and RBC velocity measurements obtained in individual SAs (S24, S25 and S26 Tables).
(PDF)

S10 Fig In vivo two-photon images of the four datasets used in the current study. *In vivo* two-photon images (top) and reconstructions of surface arteries (bottom) of the networks C57BL/6_I_ (A), C57BL/6_II_ (B), BALB/c_I_ (C) and BALB/c_II_ (D).
(PDF)

S11 FigVessel diameters before and after tuning.(A-B) Histograms of vessel diameters *d* [μm] before (A) and after (B) applying the inverse model to tune the networks. The histograms include the combined data from all four networks and blood vessels are classified into SAs (red), DAs (pink), Cs (grey) and AVs (blue). (C) Histograms of relative changes of diameters after tuning, i.e.,
$$\begin{array}{c} \text{Rel}.\text{difference}=\frac{d^{Post}-d^{Pre}}{d^{Pre}}. \end{array}$$(1)
(D-F) Visualisations of relative diameter changes in response to applying the inverse model to the network C57BL/6_I_. Arrows indicate the direction of view for panels E and F. Note that capillaries appear grey because of the perspective overlay of many transparent vessels.(PDF)

S12 Fig RBC velocity distributions. Histograms of RBC velocities *u*_*rbc*_ [*mm*/*s*] in the networks C57BL/6_I_ (A), C57BL/6_II_ (B), BALB/c_I_ (C) and BALB/c_II_ (D), classified into the vessel types SAs (red), DAs (pink), Cs (grey) and AVs (blue). The corresponding mean and median values are shown with dashed and dashed-dotted lines, respectively. Capillaries at the border of the networks, i.e., with a distance >200 μm to any DA edge, were excluded from the analysis. However, in contrast to S4 Table, velocity values for all DA and AV edge segments were included into the analysis here. Exemplary RBC velocity distributions from *in vivo* measurements are for example available in the following references: [17, 71] (DAs, AVs) and [17, 71, 74, 88–91] (Cs).
(PDF)

S13 Fig RBC flow rate distributions. Histograms of RBC flow rates *q*_*rbc*_ [fl/s] in the networks C57BL/6_I_ (A), C57BL/6_II_ (B), BALB/c_I_ (C) and BALB/c_II_ (D), classified into the vessel types SAs (red), DAs (pink), Cs (grey) and AVs (blue). The corresponding mean and median values are shown with dashed and dashed-dotted lines, respectively. Capillaries at the border of the networks, i.e., with a distance >200 μm to any DA edge, were excluded from the analysis. However, in contrast to S28 Table, flow rate values for all DA and AV edge segments were included into the analysis here. Exemplary RBC flux distributions from *in vivo* measurements are for example available in the following references: [17, 71] (DAs, AVs) and [17, 71, 74, 88, 89, 91] (Cs).
(PDF)

S1 Appendix Refinement of surface artery network. References of S1 Appendix: [3, 18, 61, 92–94].
(PDF)

S2 Appendix Blood flow model details. References of S2 Appendix: [37, 39, 70, 95].
(PDF)

S3 Appendix Inverse model details. References of S3 Appendix: [43, 70].
(PDF)

S4 Appendix Vessel elasticity model details. References of S4 Appendix: [21, 79, 81, 96, 97].
(PDF)

S1 Table *In vivo* two-photon microscopy diameter and RBC velocity measurements in surface arteries of network C57BL/6_I_ (Fig 1A) at baseline. For LMCs, diameter measurements are additionally given for the state after MCAo & LMC-dil. “x” is used if no velocity or diameter measurement was obtained in the vessel. The measurements are grouped into MCA and ACA sided SAs, and LMCs. Refer to S24, S25 and S26 Tables for measurements in other datasets.
(PDF)

S2 Table Characteristic parameters of the four networks (2x C57BL/6, 2x BALB/c) used in the present study. (PDF)

S3 Table Validation of characteristic vascular parameters with literature data. Mean ± standard deviation of diameters after tuning were calculated by considering all edge segments of the respective vessel type. Furthermore, the ranges of reported mean literature values are given in the last row. References for literature values: ^A^Length density [3, 65, 98, 99]; ^B^Volume density [3, 45–48, 99, 100]; ^C^SAs [17, 28, 101]; ^D^DAs [3, 17, 28, 84, 88, 101–103]; ^E^Cs [3, 17, 46–48, 84, 88, 99, 101–103]; ^F^AVs [3, 17, 88].
(PDF)

S4 Table Validation of characteristic velocities in different vessel types of all four networks. Mean ± standard deviation are given. Capillaries at the border of the networks, i.e., with a distance >200 μm to any DA edge, were excluded from the analysis. The average values for DAs and AVs refer to the segments of the penetrating trees closest to the cortical surface, i.e., the DA and AV root edges. The ranges of reported mean literature values are given in the last row. References for literature values: ^A^SAs [17, 26, 27, 101]; ^B^DAs [17, 37, 92, 101, 102, 104, 105]; ^C^Cs [17, 37, 74, 88–91, 101–103]; ^D^AVs [17, 71].
(PDF)

S5 TableRelative changes of mean pressure in response to MCAo and LMC-dil in MCA and ACA sided SAs of the datasets C57BL/6_I_ and C57BL/6_II_.The relative change of mean pressures after MCAo in comparison to baseline was defined as
$$\begin{array}{c} \Delta p_{rel}^{Base\to MCAo}=\frac{\text{mean}(p^{MCAo})-\text{mean}(p^{Base})}{\text{mean}(p^{Base})}, \end{array}$$(2)
where *p*^*Base*^ and *p*^*MCAo*^ are the pressure values at baseline and after MCAo, respectively. Analogously, the superscript MCAo → MCAo & LMC-dil denotes the relative change from MCAo to MCAo & LMC-dil. Refer to S13 Table for results after LMC/SA/DA-dil.(PDF)

S6 TableRelative changes of mean flow rate in response to MCAo and LMC-dil in SAs of the datasets C57BL/6_I_ and C57BL/6_II_.The relative change of mean flow rate after MCAo in comparison to baseline was defined as
$$\begin{array}{c} \Delta q_{rel}^{Base\to MCAo}=\frac{\text{mean}(q^{MCAo})-\text{mean}(q^{Base})}{\text{mean}(q^{Base})}, \end{array}$$(3)
where *q*^*Base*^ and *q*^*MCAo*^ are the flow rates at baseline and after MCAo. Analogously, the superscripts MCAo → MCAo & LMC-dil and Base → MCAo & LMC-dil denote relative changes from MCAo to MCAo & LMC-dil and from baseline to MCAo & LMC-dil, respectively. Refer to S14 Table for results after LMC/SA/DA-dil.(PDF)

S7 TableRelative changes of integral flow rate after MCAo and LMC-dil in DAs of the datasets C57BL/6_I_ and C57BL/6_II_.The relative change of integral flow rate after MCAo in comparison to baseline was defined as
$$\begin{array}{c} \Delta Q_{rel}^{Base\to MCAo}=\frac{\text{sum}(q^{MCAo})-\text{sum}(q^{Base})}{\text{sum}(q^{Base})}, \end{array}$$(4)
where *q*^*Base*^ and *q*^*MCAo*^ are the flow rates at baseline and after MCAo. Analogously, the superscripts MCAo → MCAo & LMC-dil and Base → MCAo & LMC-dil denote relative changes from MCAo to MCAo & LMC-dil and from baseline to MCAo & LMC-dil, respectively. Refer to S15 Table for results after LMC/SA/DA-dil.(PDF)

S8 TableRelative changes of integral flow rate after MCAo and LMC-dil in Cs of the datasets C57BL/6_I_ and C57BL/6_II_.Refer to the caption of S7 Table for the definitions of $\Delta Q_{rel}^{Base\to MCAo}$, $\Delta Q_{rel}^{MCAo\to MCAo\&LMC-dil}$ and $\Delta Q_{rel}^{Base\to MCAo\&LMC-dil}$. Refer to S16 Table for results after LMC/SA/DA-dil.(PDF)

S9 Table Relative changes of mean flow rate in SAs (definition in S6 Table) in response to MCAo and MCAo & LMC-dil for different number of LMCs. 〈…〉 is used to refer to average values of all four datasets. The results are consistent with the bars in Fig 5B. Refer to S18 Table for results after LMC/SA/DA-dil.
(PDF)

S10 Table Relative changes of mean pressure in SAs (definition in S5 Table) in response to MCAo and MCAo & LMC-dil for different number of LMCs. 〈…〉 is used to refer to average values of all four datasets. Refer to S17 Table for results after LMC/SA/DA-dil.
(PDF)

S11 Table Relative changes of integral flow rate in DAs (definition in S7 Table) in response to MCAo and MCA & LMC-dil for different number of LMCs. 〈…〉 is used to refer to average values computed over all four datasets. The results are consistent with the bars in Fig 5C and 5D. Refer to S19 Table for results after LMC/SA/DA-dil.
(PDF)

S12 Table Relative changes of integral flow rate in Cs (definition in S8 Table) in response to MCAo and MCAo & LMC-dil for different number of LMCs. 〈…〉 is used to refer to average values computed over all four datasets. The results are consistent with the bars in Fig 5E and 5F. Refer to S20 Table for results after LMC/SA/DA-dil.
(PDF)

S13 Table Relative changes of mean pressure (definition in S5 Table) in response to MCAo & LMC/SA/DA-dil in MCA and ACA sided SAs of the datasets C57BL/6_I_ and C57BL/6_II_. (PDF)

S14 Table Relative changes of mean flow rate (definition in S6 Table) in response to MCAo & LMC/SA/DA-dil in SAs of the datasets C57BL/6_I_ and C57BL/6_II_ in comparison to Base and MCAo. (PDF)

S15 Table Relative changes of integral flow rate (definition in S7 Table) after MCAo & LMC/SA/DA-dil in DAs of the datasets C57BL/6_I_ and C57BL/6_II_ in comparison to Base and MCAo. (PDF)

S16 Table Relative changes of integral flow rate (definition in S7 Table) after MCAo & LMC/SA/DA-dil in Cs of the datasets C57BL/6_I_ and C57BL/6_II_ in comparison to Base and MCAo. (PDF)

S17 Table Relative changes of mean pressure in SAs (definition in S5 Table) in response to MCAo & LMC/SA/DA-dil for different number of LMCs. 〈…〉 is used to refer to average values computed over all four datasets.
(PDF)

S18 Table Relative changes of mean flow rate in SAs (definition in S6 Table) in response to MCAo & LMC/SA/DA-dil for different number of LMCs in comparison to Base and MCAo. 〈…〉 is used to refer to average values computed over all four datasets.
(PDF)

S19 Table Relative changes of integral flow rate in DAs (definition in S7 Table) in response to MCAo & LMC/SA/DA-dil for different number of LMCs. 〈…〉 is used to refer to average values computed over all four datasets.
(PDF)

S20 Table Relative changes of integral flow rate in Cs (definition in S7 Table) in response to MCAo & LMC/SA/DA-dil for different number of LMCs. 〈…〉 is used to refer to average values computed over all four datasets.
(PDF)

S21 Table Comparison of relative changes of mean flow rates (definition in S6 Table) in SAs after MCAo with and without taking the elasticity of blood vessels into account. (PDF)

S22 Table Comparison of relative changes of integral flow rates (definition in S7 Table) in DAs after MCAo with and without taking the elasticity of blood vessels into account. (PDF)

S23 Table Comparison of relative changes of integral flow rates (definition in S7 Table) in Cs after MCAo with and without taking the elasticity of blood vessels into account. (PDF)

S24 Table *In vivo* two-photon microscopy diameter and RBC velocity measurements in pial arteries of network BALB/c_I_ (S9(A) Fig) at baseline. “x” is used if no velocity or diameter measurement was obtained in the vessel. The measurements are grouped into MCA and ACA sided SAs.
(PDF)

S25 Table *In vivo* two-photon microscopy diameter measurements in pial arteries of network C57BL/6_II_ (S9(C) Fig) at baseline. The measurements are grouped into MCA and ACA sided SAs.
(PDF)

S26 Table *In vivo* two-photon microscopy diameter measurements in pial arteries of network BALB/c_II_ (S9(B) Fig) at baseline. The measurements are grouped into MCA and ACA sided SAs.
(PDF)

S27 Table Comparison of simulated cerebral blood flow. The average blood flow per surface area and tissue volume were determined based on the total blood flow that enters the network and the estimated surface area of the network, which was calculated from the Voronoi polygons shown in Fig A panel B in S1 Appendix. Based on the average blood flow per tissue volume, cerebral blood flow per mass was computed assuming a tissue density of 1046 kg/m^3^ [106]. References for CBF literature values: ^A^CBF [107–111].
(PDF)

S28 Table Validation of characteristic RBC flow rates in different vessel types of all four networks. Mean ± standard deviation are given. Capillaries at the border of the networks, i.e., with a distance >200 μm to any DA edge, were excluded from the analysis. The average values for DAs and AVs refer to the segments of the penetrating trees closest to the cortical surface, i.e., the DA and AV root edges. The ranges of reported mean literature values are given in the last row. References for literature values: ^A^DAs [17, 37, 71, 92]; ^B^Cs [17, 37, 71, 88, 89, 91]; ^C^AVs [17, 71].
(PDF)

S29 TableValidation of wall shear stress (WSS) in different vessel types of all four networks.The wall shear stress in each vessel was estimated based on the flow rate *q*_*ij*_, the diameter *d*_*ij*_ and the effective viscosity μ_*eff*,*ij*_ = μ_*p*_ μ_*rel*, *ij*_ (please refer to S2 Appendix for the nomenclature) by assuming a parabolic velocity profile, i.e.,
$$\begin{array}{c} WSS_{ij}=\frac{32\;q_{ij}\;\mu_{eff,ij}}{\pi d_{ij}^{3}}. \end{array}$$(5)
Mean ± standard deviation are given for all vessel types. Capillaries at the border of the networks, i.e., with a distance >200 μm to any DA edge, were excluded from the analysis. The average values for DAs and AVs refer to the segments of the penetrating trees closest to the cortical surface, i.e., the DA and AV root edges. The ranges of reported mean literature values obtained for the mesentery [112–114] and from a simulation study [115] are given in the last row. References for literature values: ^A^SAs & DAs [112–115]; ^B^Cs [112, 114, 115]; ^C^AVs [112–115].(PDF)
