# Supplementary figures and images for: The role of leptomeningeal collaterals in redistributing blood flow during stroke
Source: PLoS Comput Biol. 2023 Oct 23;19(10):e1011496. doi: 10.1371/journal.pcbi.1011496 (PMC10621965; doi:10.1371/journal.pcbi.1011496)

A

Rel. flow rate change in SAs [-]  
Base → MCAo

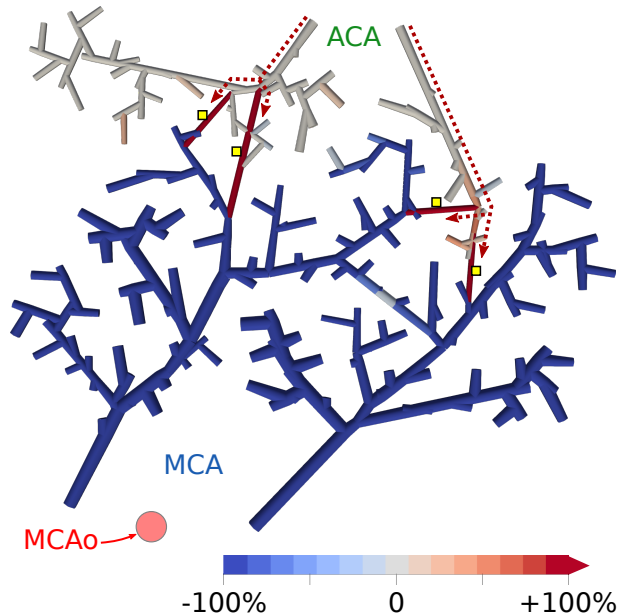

B

Rel. flow rate change in SAs [-]  
MCAo → MCAo & LMC-dil

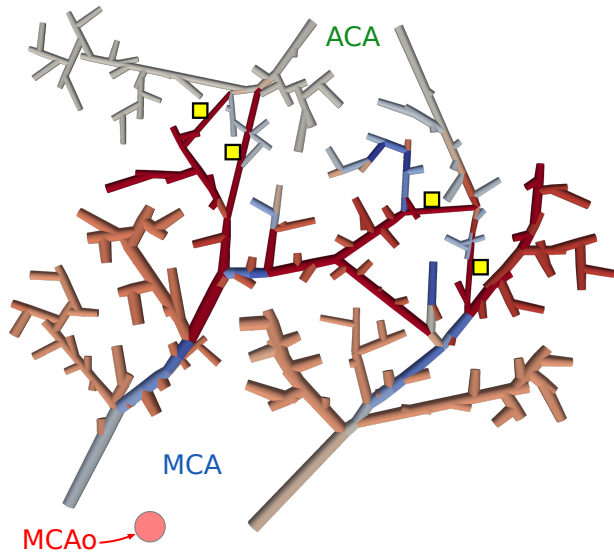

Supplement: S1 Fig — Relative changes of blood flow rates in individual SAs of the network C57BL/6II from Base to MCAo (A) and MCAo to MCAo & LMC-dil (B). The yellow squares indicate the locations of the LMCs. Supplement to Fig 3A and 3B. Refer to S7(A) Fig for results after LMC/SA/DA-dil. (PDF) [file pcbi.1011496.s001.pdf]

**A**

Direction changes in SAs [-]  
Base → MCAo

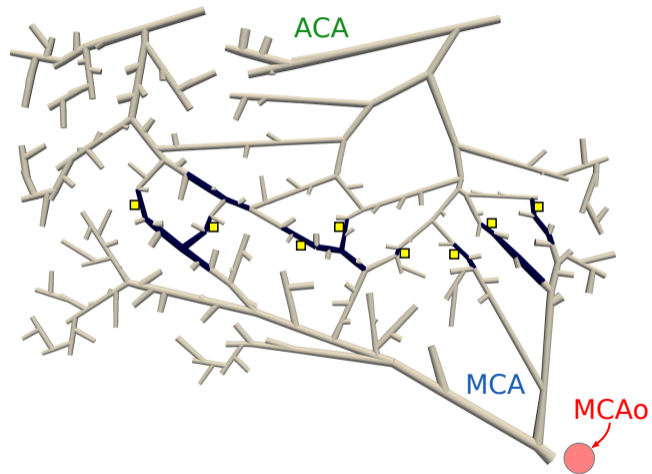**B**

Direction changes in SAs [-]  
Base → MCAo

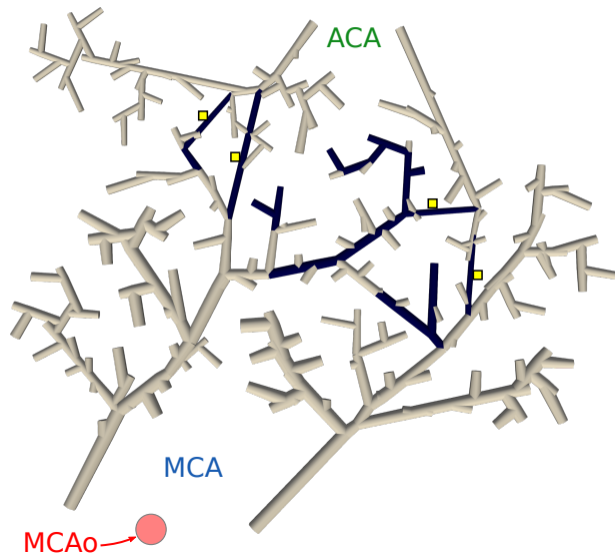

Supplement: S2 Fig — Edges with direction changes (dark blue) in response to MCAo for the networks C57BL/6I (A) and C57BL/6II (B). The yellow squares indicate the locations of the LMCs. Supplement to Fig 3. (PDF) [file pcbi.1011496.s002.pdf]

**A**

Rel. flow rate change in DAs [-]  
Base → MCAo

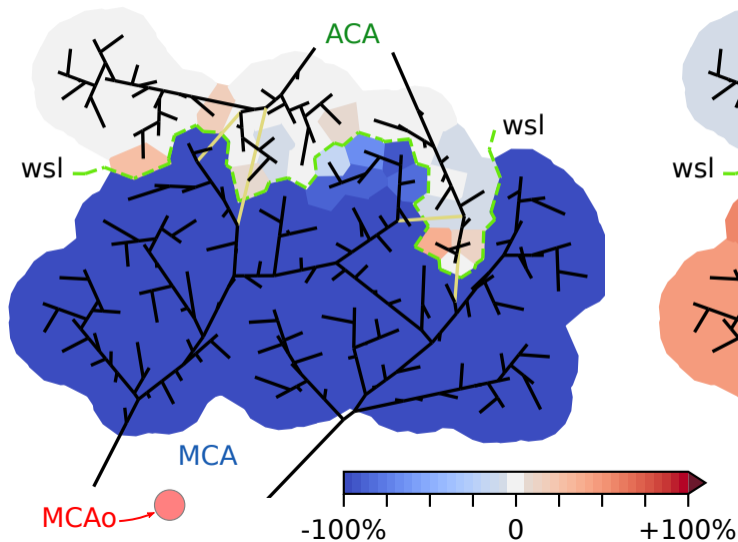**B**

Rel. flow rate change in DAs [-]  
MCAo → MCAo & LMC-dil

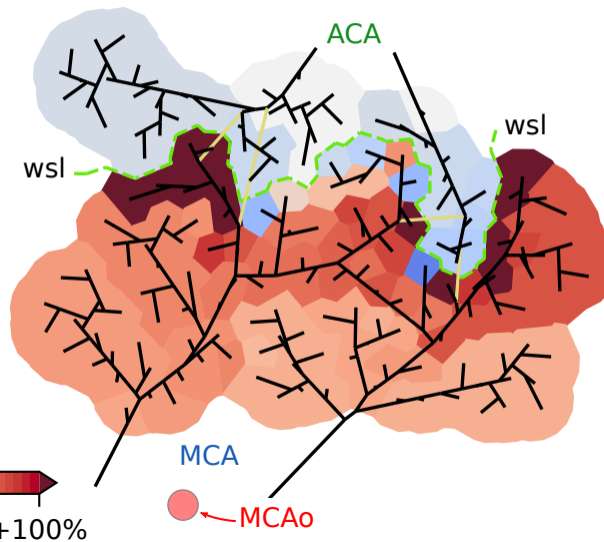

Supplement: S3 Fig — Relative changes of blood flow rates in DAs of the network C57BL/6II from Base to MCAo (A) and MCAo to MCAo & LMC-dil (B). Supplement to Fig 4A and 4B. Refer to S8 Fig for results after LMC/SA/DA-dil. (PDF) [file pcbi.1011496.s003.pdf]

**A**

Diameter changes in SAs [-]  
Base → MCAo

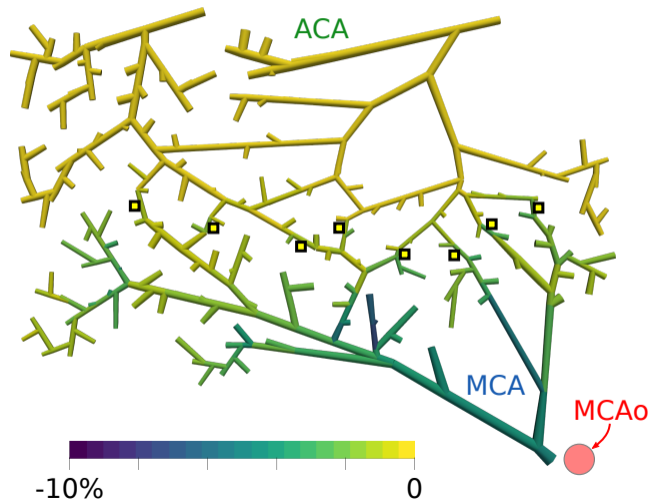**B**

Diameter changes in SAs [-]  
Base → MCAo

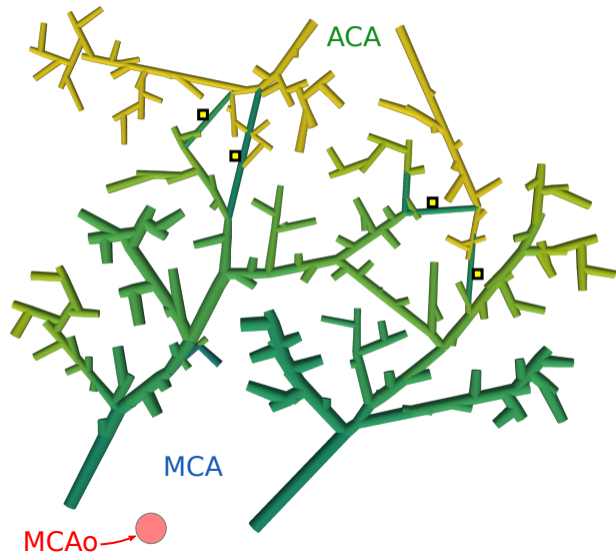

Supplement: S4 Fig — Relative changes of SA diameters in response to MCAo in the networks C57BL/6I (A) and C57BL/6II (B). (PDF) [file pcbi.1011496.s004.pdf]

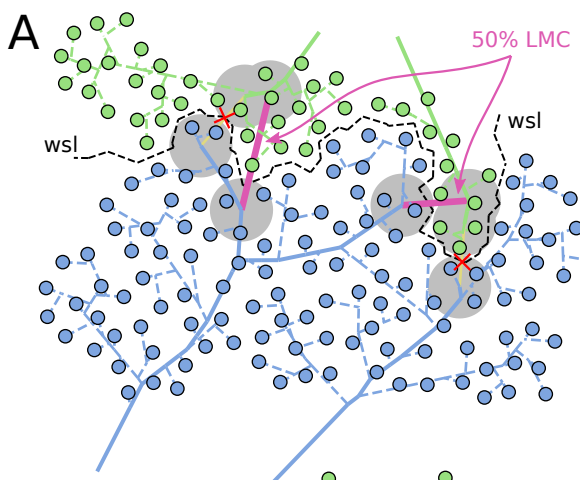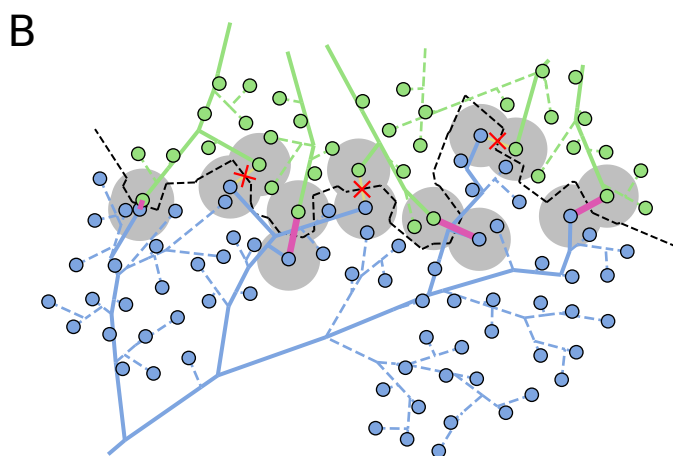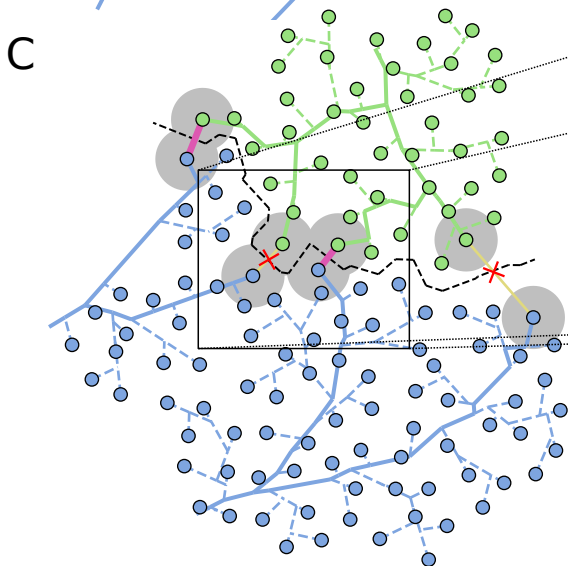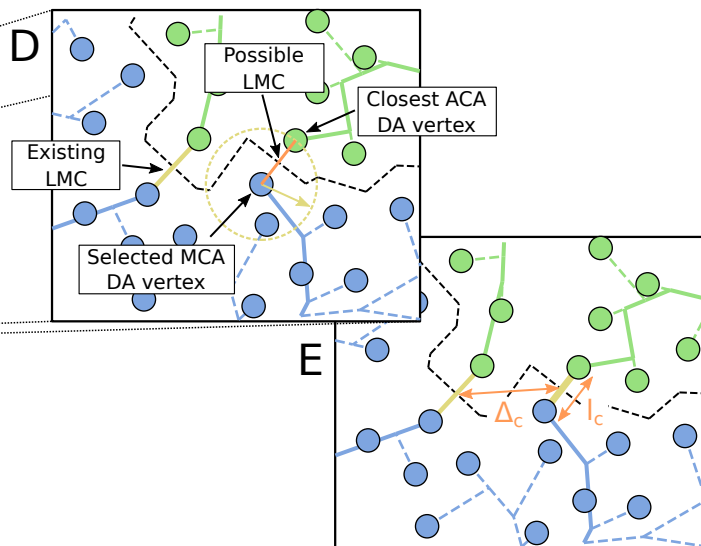

Supplement: S5 Fig — Maps of the pial networks C57BL/6II (A), BALB/cI (B) and BALB/cII (C), and visualisation of the three scenarios with many, few and no LMCs. Supplement to Fig 5A. To define the number of added LMCs for the 100% LMC scenarios of the BALB/c datasets, the LMC density along the watershed line was computed from the two C57BL/6 datasets. The goal was then to obtain the same overall LMC density along the watershed line for the BALB/c networks. This was done with a sequential procedure by randomly selecting MCA DA vertices at the watershed line and connecting them to the closest DA vertices in the ACA territory (D). The sampled LMCs were then accepted or rejected based on two criteria derived from the C57BL/6 datasets: 1) The distance to already existing LMCs Δc was larger than 310 μm and 2) the maximum LMC length lc was 1000 μm (E). As for the C57BL/6 networks, the 50% LMC scenario for BALB/c networks was defined by randomly removing LMCs. (PDF) [file pcbi.1011496.s005.pdf]

**A**

Rel. pressure change in SAs [-]  
Base → MCAo

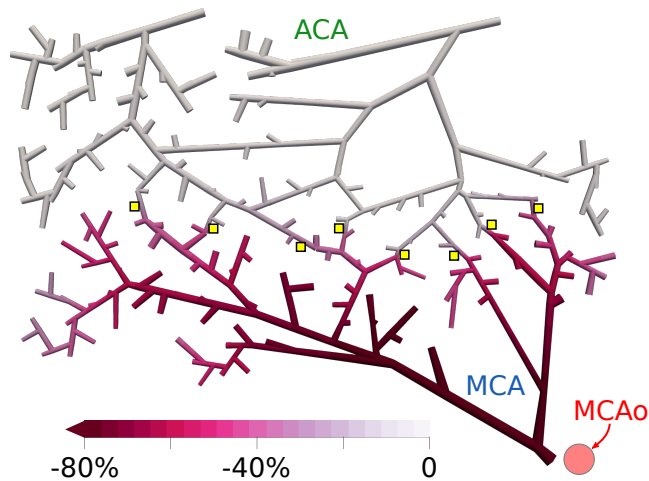**B**

Rel. pressure change in SAs [-]  
MCAo → MCAo & LMC-dil

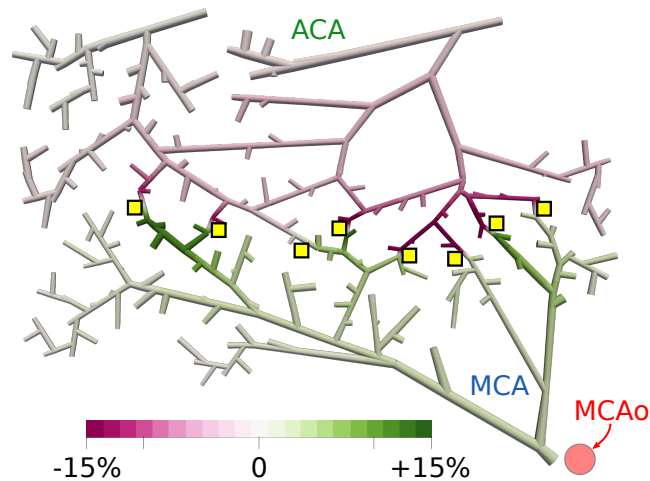**C**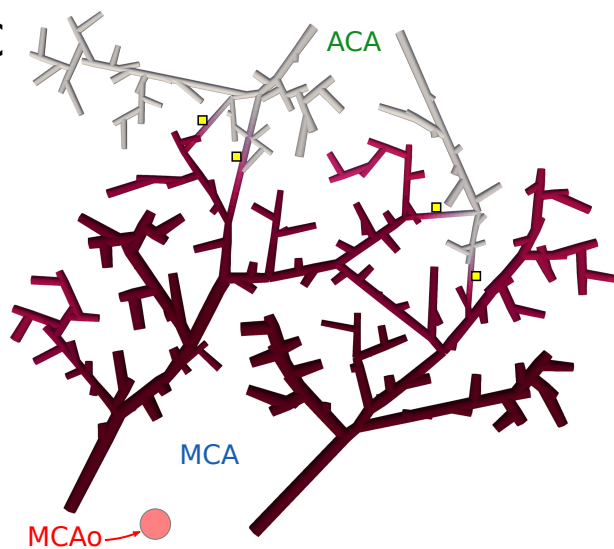**D**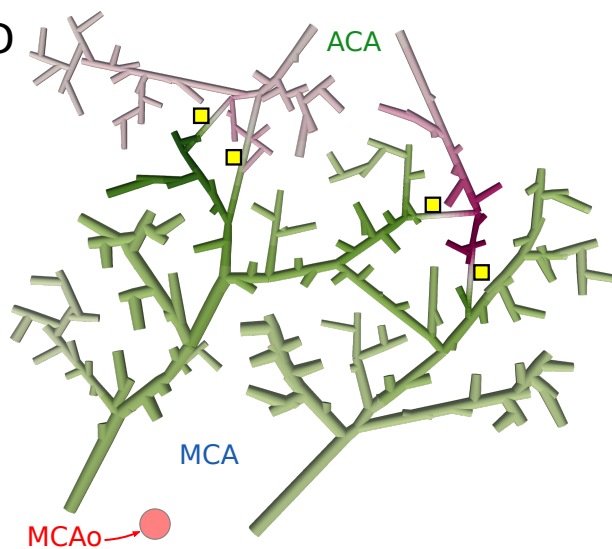

Supplement: S6 Fig — Relative changes of pressures in SAs of the network C57BL/6I from Base to MCAo (A) and MCAo to MCAo & LMC-dil (B). The results for the network C57BL/6II are shown in panels (C) and (D), respectively. Refer to S7(B) and S7(C) Fig for results after LMC/SA/DA-dil. (PDF) [file pcbi.1011496.s006.pdf]

Rel. flow rate change in DAs [-]  
MCAo & LMC-dil → MCAo & LMC/SA/DA-dil

A

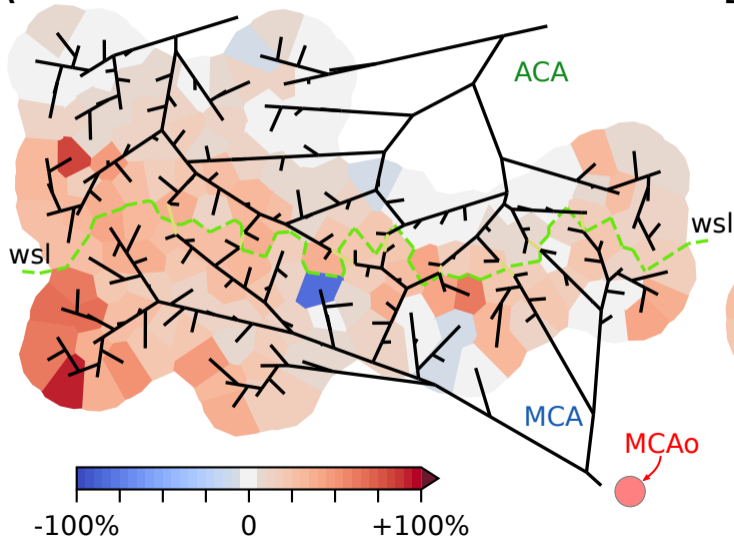

B

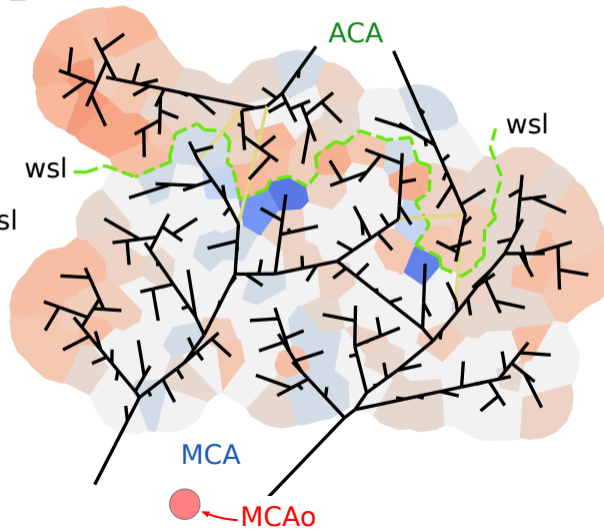

Supplement: S8 Fig — Relative changes of blood flow rates in DAs from MCAo & LMC-dil to MCAo & LMC/SA/DA-dil in the networks C57BL/6I (A) and C57BL/6II (B). (PDF) [file pcbi.1011496.s008.pdf]

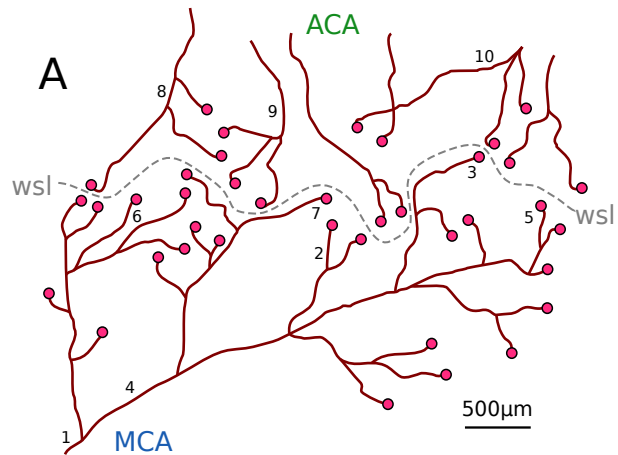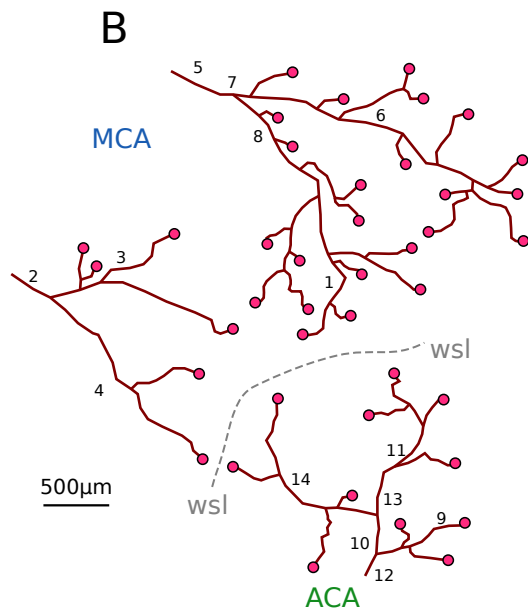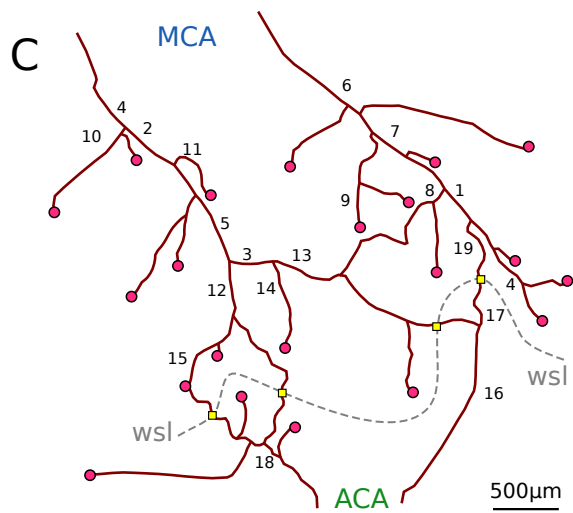

Supplement: S9 Fig — Experimentally acquired reconstructions of the surface artery (SA) networks BALB/cI (A), BALB/cII (B) and C57BL/6II (C). The numbers refer to diameter and RBC velocity measurements obtained in individual SAs (S24, S25 and S26 Tables). (PDF) [file pcbi.1011496.s009.pdf]

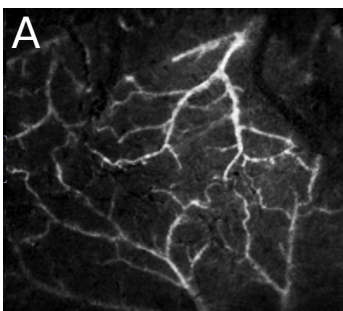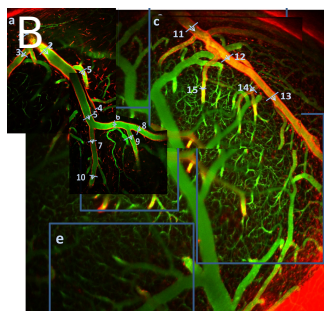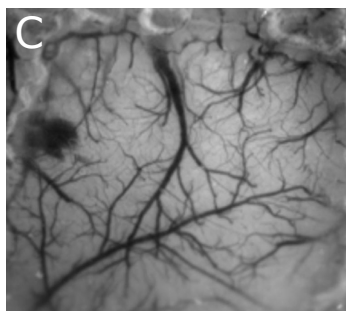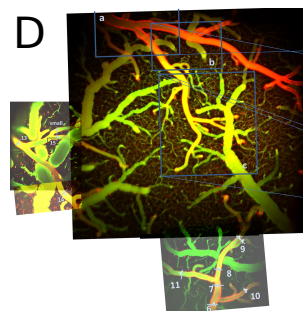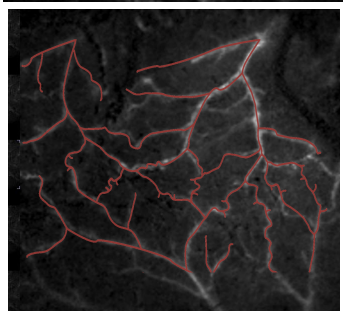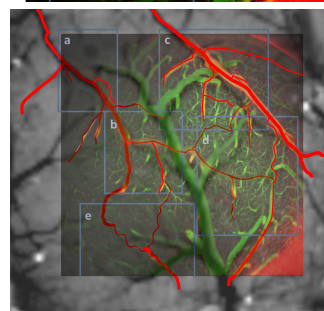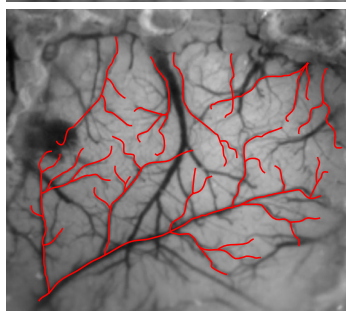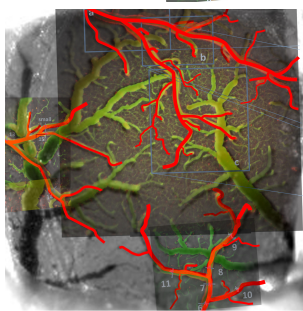

Supplement: S10 Fig — In vivo two-photon images (top) and reconstructions of surface arteries (bottom) of the networks C57BL/6I (A), C57BL/6II (B), BALB/cI (C) and BALB/cII (D). (PDF) [file pcbi.1011496.s010.pdf]

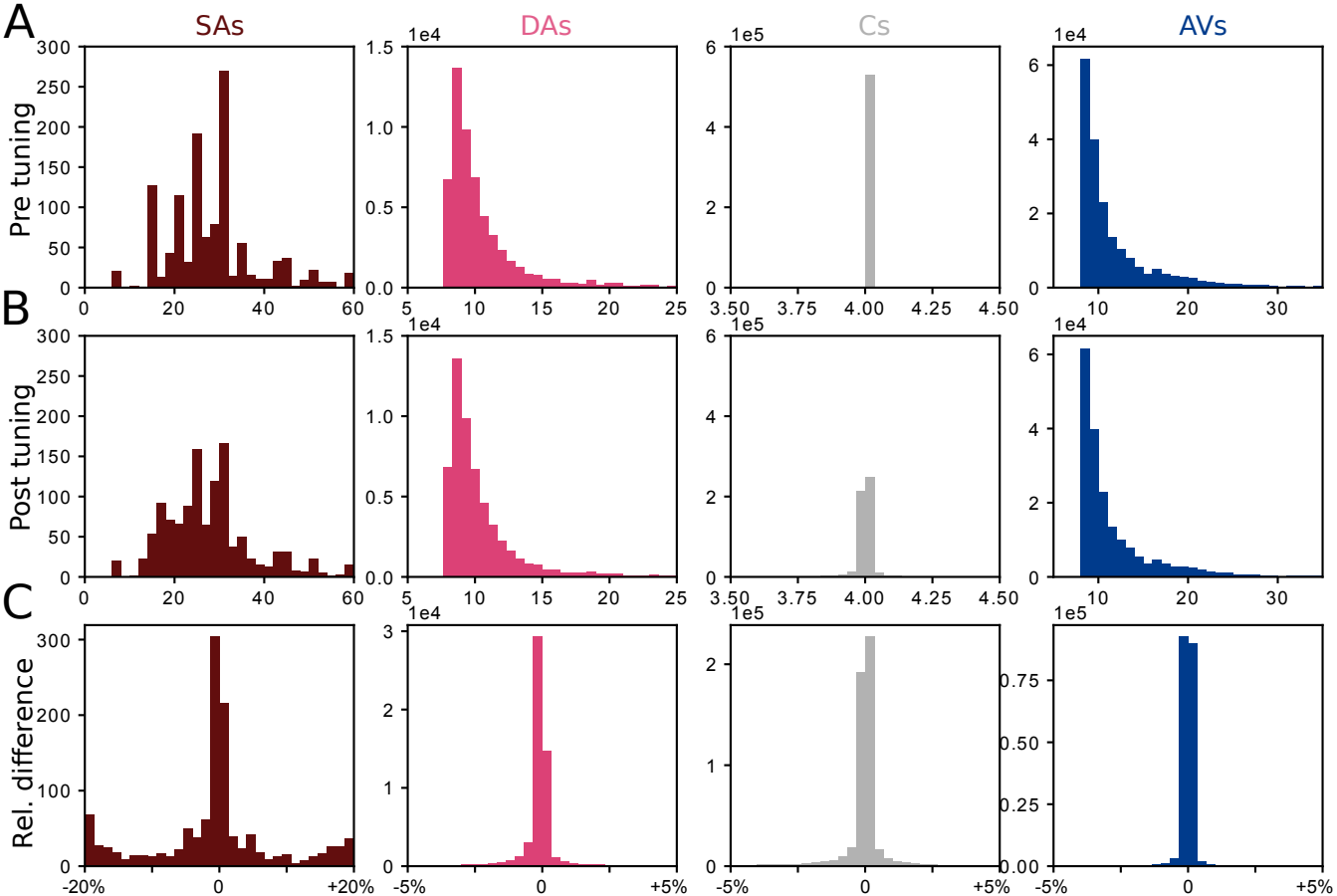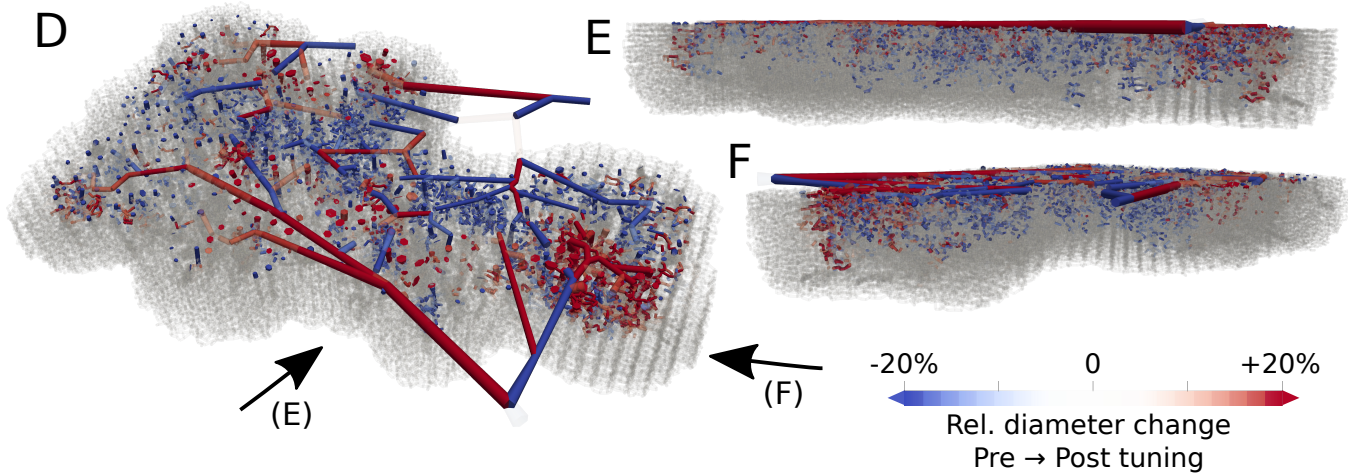

Supplement: S11 Fig — (A-B) Histograms of vessel diameters d [μm] before (A) and after (B) applying the inverse model to tune the networks. The histograms include the combined data from all four networks and blood vessels are classified into SAs (red), DAs (pink), Cs (grey) and AVs (blue). (C) Histograms of relative changes of diameters after tuning, i.e., Rel.difference=dPost-dPredPre.(1) (D-F) Visualisations of relative diameter changes in response to applying the inverse model to the network C57BL/6I. Arrows indicate the direction of view for panels E and F. Note that capillaries appear grey because of the perspective overlay of many transparent vessels. (PDF) [file pcbi.1011496.s011.pdf]

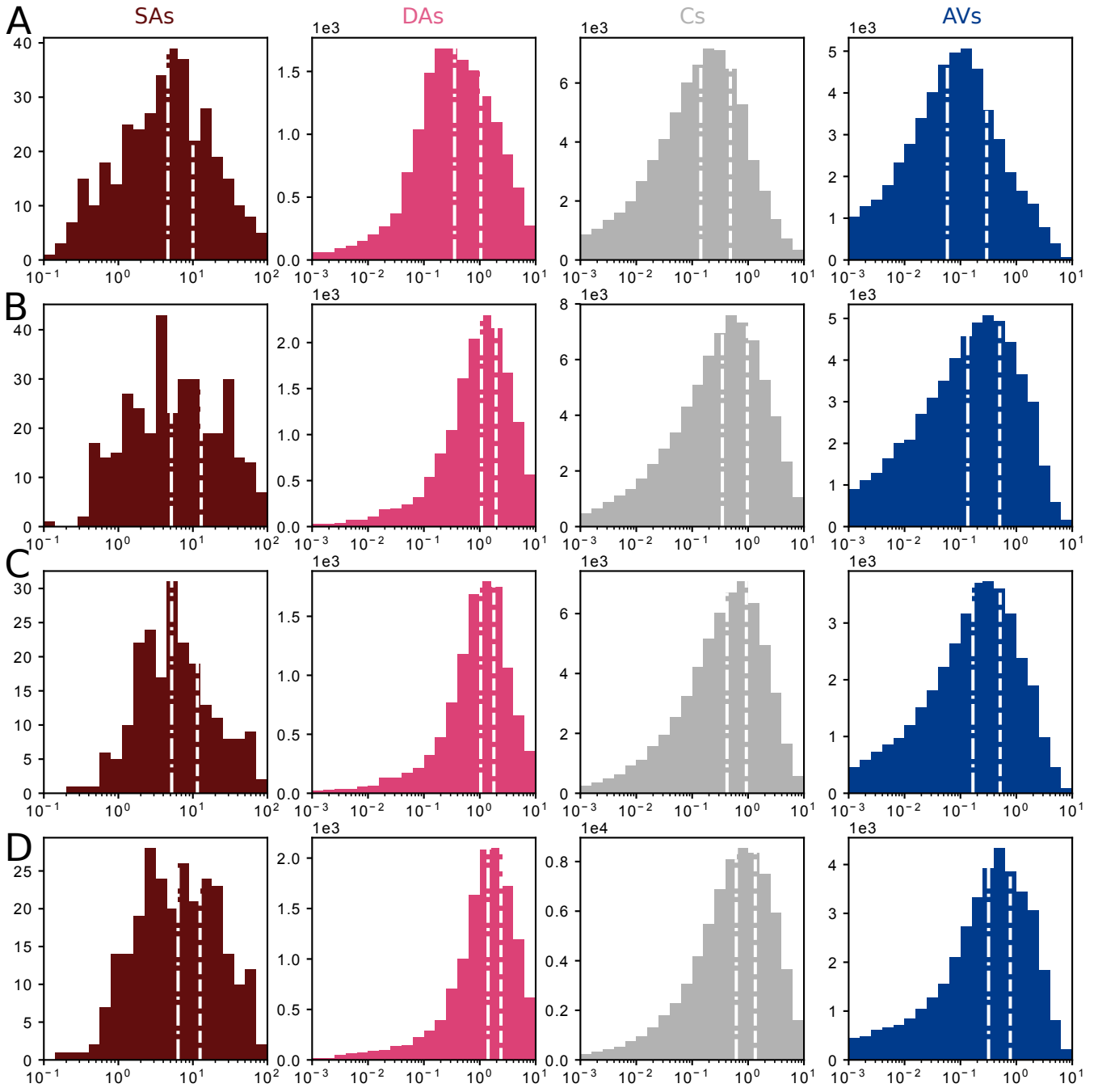

Supplement: S12 Fig — Histograms of RBC velocities urbc [mm/s] in the networks C57BL/6I (A), C57BL/6II (B), BALB/cI (C) and BALB/cII (D), classified into the vessel types SAs (red), DAs (pink), Cs (grey) and AVs (blue). The corresponding mean and median values are shown with dashed and dashed-dotted lines, respectively. Capillaries at the border of the networks, i.e., with a distance >200 μm to any DA edge, were excluded from the analysis. However, in contrast to S4 Table, velocity values for all DA and AV edge segments were included into the analysis here. Exemplary RBC velocity distributions from in vivo measurements are for example available in the following references: [17, 71] (DAs, AVs) and [17, 71, 74, 88–91] (Cs). (PDF) [file pcbi.1011496.s012.pdf]

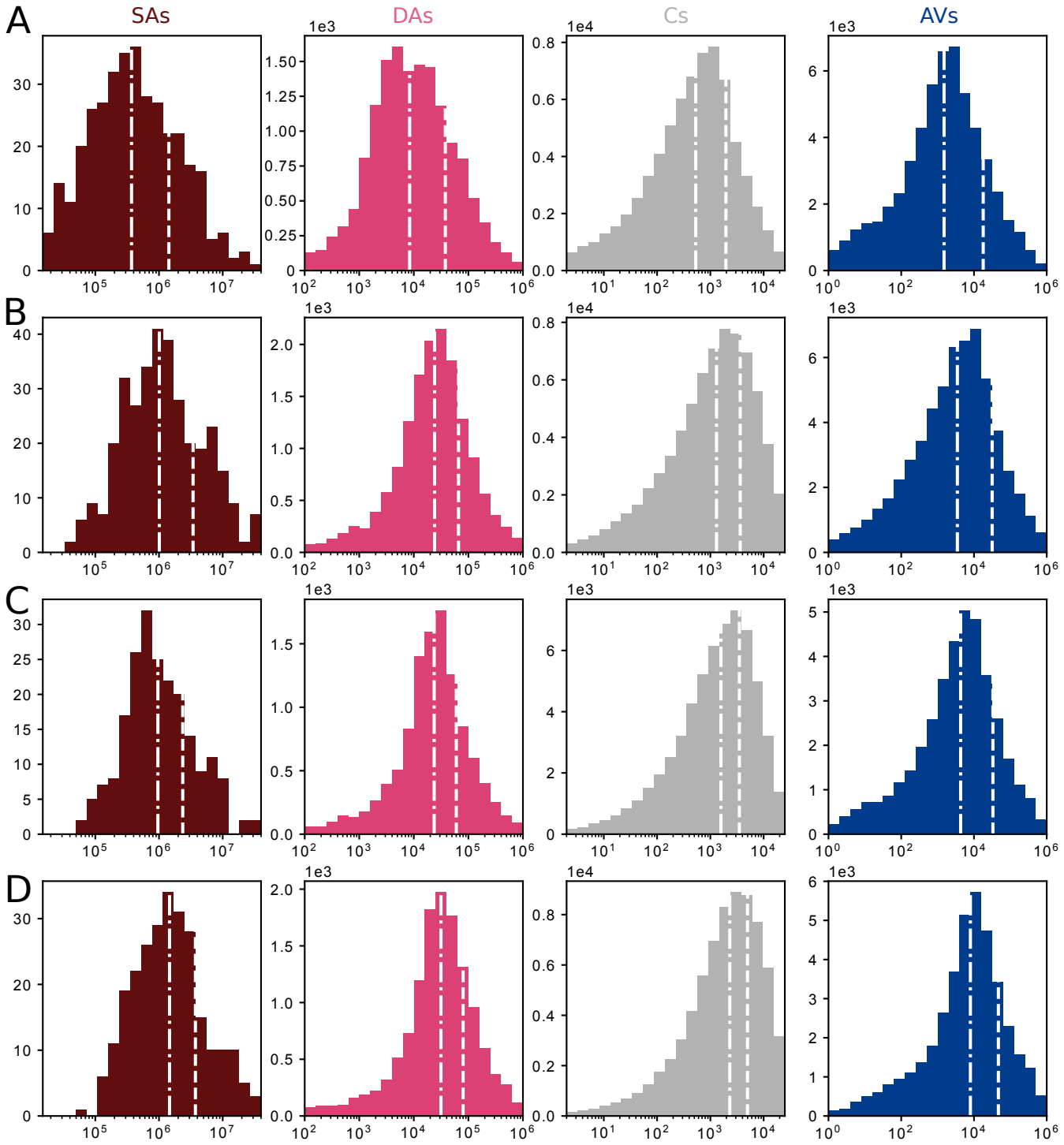

Supplement: S13 Fig — Histograms of RBC flow rates qrbc [fl/s] in the networks C57BL/6I (A), C57BL/6II (B), BALB/cI (C) and BALB/cII (D), classified into the vessel types SAs (red), DAs (pink), Cs (grey) and AVs (blue). The corresponding mean and median values are shown with dashed and dashed-dotted lines, respectively. Capillaries at the border of the networks, i.e., with a distance >200 μm to any DA edge, were excluded from the analysis. However, in contrast to S28 Table, flow rate values for all DA and AV edge segments were included into the analysis here. Exemplary RBC flux distributions from in vivo measurements are for example available in the following references: [17, 71] (DAs, AVs) and [17, 71, 74, 88, 89, 91] (Cs). (PDF) [file pcbi.1011496.s013.pdf]
