# Supplementary material for: The role of leptomeningeal collaterals in redistributing blood flow during stroke
Source: PLoS Comput Biol. 2023 Oct 23;19(10):e1011496. doi: 10.1371/journal.pcbi.1011496 (PMC10621965; doi:10.1371/journal.pcbi.1011496)
Supplement: S7 Fig — (A) Relative changes of flow rates in SAs of the network C57BL/6II from MCAo & LMC-dil to MCAo & LMC/SA/DA-dil. (B-C) The corresponding pressure changes are shown in panels (B) and (C) for both networks C57BL/6I and C57BL/6II, respectively. (PDF) [file pcbi.1011496.s007.pdf]

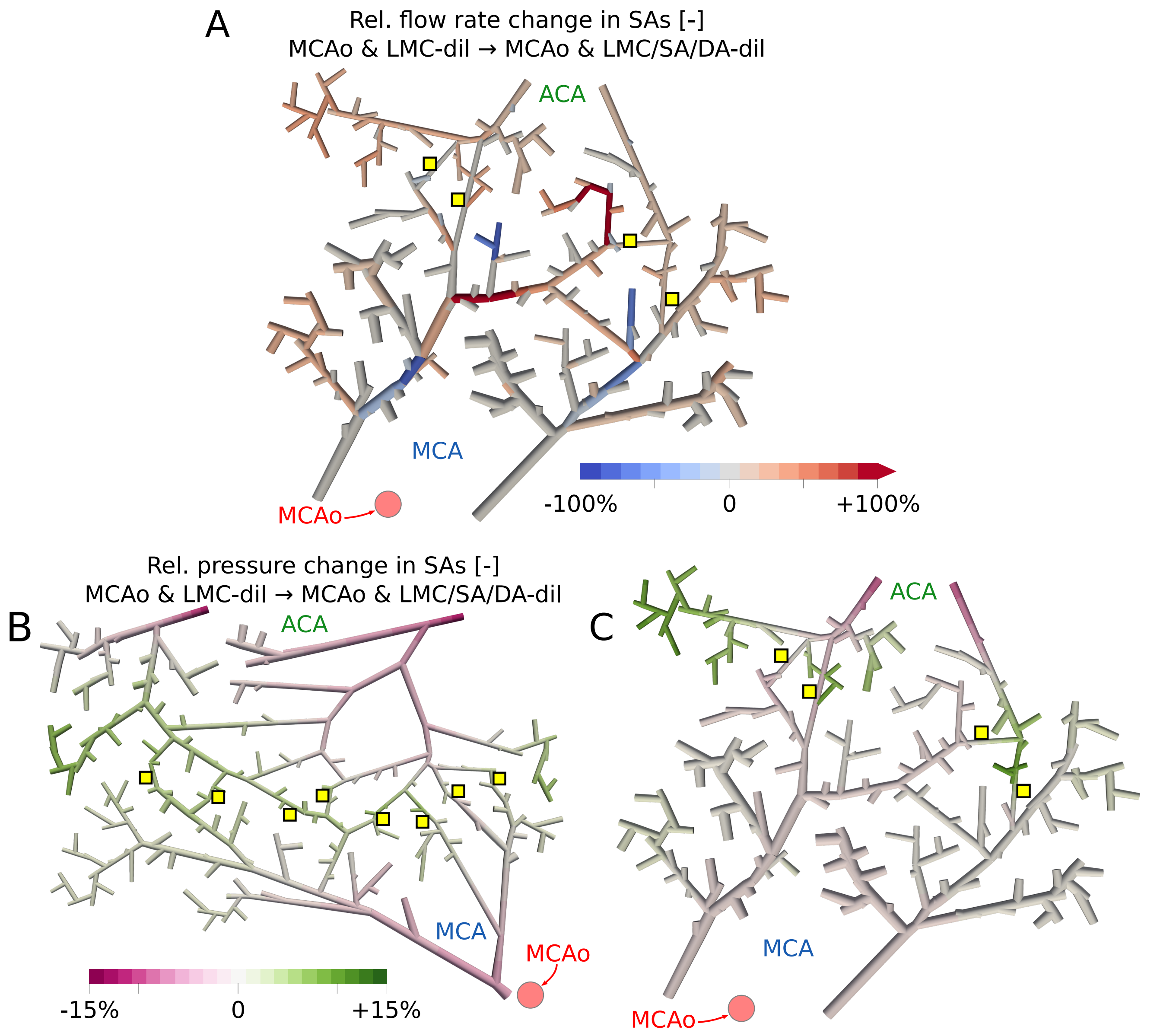

A
Rel. flow rate change in SAs [-]
MCAo & LMC-dil → MCAo & LMC/SA/DA-dil
ACA
MCA
MCAo
-100%
0
+100%
Rel. pressure change in SAs [-]
MCAo & LMC-dil → MCAo & LMC/SA/DA-dil
B
ACA
MCA
MCAo
-15%
0
+15%
C
ACA
MCA
MCAo
