# Supplementary material for: The role of leptomeningeal collaterals in redistributing blood flow during stroke
Source: PLoS Comput Biol. 2023 Oct 23;19(10):e1011496. doi: 10.1371/journal.pcbi.1011496 (PMC10621965; doi:10.1371/journal.pcbi.1011496)
Supplement: S1 Appendix — References of S1 Appendix: [3, 18, 61, 92–94]. (PDF) [file pcbi.1011496.s014.pdf]

# S1 Appendix. Refinement of surface artery network.

The reconstruction of SAs provided the locations of only a few DA roots (Fig 1A) since not all DA roots could be detected by the *in vivo* imaging approach employed for this study. Consequently, we artificially increased the DA root density to match typical values for both mouse strains by using an acceptance-rejection sampling approach. Therefore, the geometrical locations of all available DA roots were used to compute the Voronoi tessellation and estimate the approximate feeding areas per DA root (Fig A panel A in S1 Appendix). The coordinates of a possible additional DA root location were then generated with a uniform random number generator. The new DA root sample point was accepted if (a) it was located within the area of one of the original Voronoi polygons shown in Fig A panel A in S1 Appendix, (b) if the surface area of the current polygon was larger than a pre-defined threshold value $A_{lim}$ and (c) if its position was at a certain minimum distance to already existing DA roots (Min DA-DA in Table A in S1 Appendix). If one of these conditions was violated, the DA root candidate was rejected and a new point was sampled. This procedure was repeated until the desired target density was matched. The parameter $A_{lim}$ varies slightly for different networks and was chosen as large as possible, while ensuring that the prescribed average target density was matched.

**Table A.** Target DA tree densities, corresponding average feeding areas and the employed minimal distances between penetrating trees for C57BL/6 and BALB/c networks. The average feeding area corresponds to the reciprocal value of the target density and was used to derive the minimum distances between two DAs (Min. DA-DA). More precisely, Min. DA-DA was defined as 75% of the side length of a square with an area identical to the average feeding area, i.e., Min. DA-DA $= 0.75 \cdot \sqrt{\text{Avg. feeding area}}$. Since AVs are arranged around DA trees, the Min. DA-AV distance corresponds to 50% of Min. DA-DA. The minimal distance between two AV trees (Min. AV-AV) was derived analogously based on the desired target AV density, i.e., Min. AV-AV $= 0.75 \cdot \sqrt{\frac{\text{Avg. feeding area}}{3}}$ for an AV:DA ratio of 3:1.

| | Target density | Avg. feeding area | Min. DA-DA | Min. DA-AV | Min. AV-AV |
|---|---|---|---|---|---|
| C57BL/6 | 13.4 DAs/mm$^2$ | 0.075 mm$^2$ | 200 µm | 100 µm | 120 µm |
| BALB/c | 8.9 DAs/mm$^2$ | 0.112 mm$^2$ | 250 µm | 125 µm | 145 µm |

Generally, the variability of DA densities is large across different brain regions [1], mouse strains and individual animals [2]. In the current work, we assumed a uniform target density for each mouse strain (Table A in S1 Appendix). The target values were obtained experimentally by using laser scanning microscopy, i.e., by counting the number of DA trees in four mouse brains (2x C57BL/6 and 2x BALB/c) and computing average values for both strains. Mouse brains cleared and imaged from an ongoing project were used [3]. Briefly, Light Sheet microscopy (meso-SPIM, version V5) [4] was used to scan modified iDISCO cleared [5] and alpha-smooth muscle actin stained mouse brains. Manual quantification of LMCs and penetrating artery density was performed using Fiji (Image J, 1.8.0_172 64 bit). The resulting densities are in line with data from previous experimental studies [1, 2, 6].

In Fig A panel B in S1 Appendix, the final locations of all original and newly added DA roots are shown together with the corresponding Voronoi polygons. The total number of DA roots now matches the desired target density, and they are distributed such that all DAs are well separated from each other. Due to the high flexibility of this acceptance-rejection sampling approach, the method could easily be extended to not only match overall mean DA densities, but to incorporate any additional knowledge on

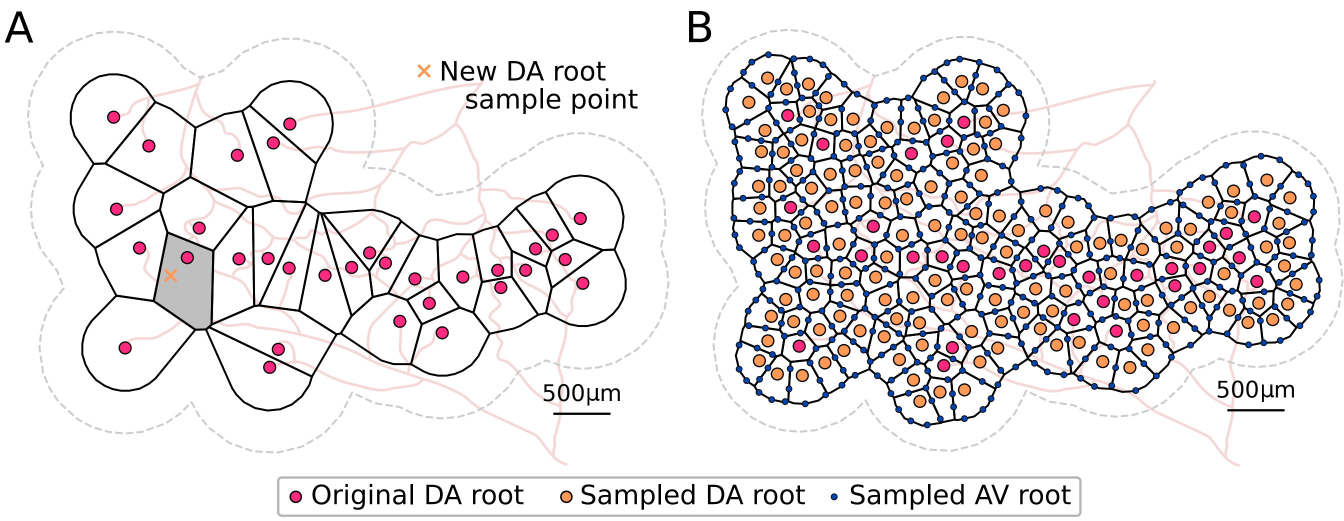


**Fig A.** Refinement of DA density. (A) Voronoi tessellation for the original DA roots detected in the experiments (red dots). After sampling a new possible location for an additional DA root point (orange cross), the corresponding Voronoi polygon was identified and the point was accepted or rejected based on predefined criteria. (B) Distribution of DA roots after refinement with the newly added DA root points visualised as orange dots. The locations of AV root points (blue dots) were sampled directly onto the edges of the Voronoi polygons.

the distribution of DA roots in the vasculature, e.g. the probability distribution of feeding areas or information on the local variability of DA densities [1, 2].

In the last step the newly sampled DA roots were connected to the pial network. This is done by randomly selecting a DA root that was yet unconnected, and assigning it to the nearest SA. A new vessel was then added between the DA root and a newly created connection point on the SA. The procedure was repeated until all DA roots were connected to the SA network, as seen in Fig 1B. This approach ensures that the backbone of the pial network remains unchanged and that the sampled DAs are added as offshoots from the backbone.

Since the experiments (Fig 1A) only provided information on the topology of SAs and the position of a few DA roots, the exact locations of AV roots at the cortical surface, i.e., the location where AV trees are connected to pial veins, were unknown. Therefore, we distributed AV roots such that they are positioned around DA roots [6], which was achieved by sampling their locations directly on the edges of the Voronoi polygons (Fig A panel B in S1 Appendix). Here, the goal was to obtain an AV:DA ratio of approximately 3:1 [6], while ensuring certain minimum distances to other already existing DA or AV roots (Table A in S1 Appendix). In contrast to the DA roots, the sampled AV roots were not connected to a network of pial veins and remain freely distributed (Fig 1B).

# References of S1 Appendix

1. Blinder P, Shih AY, Rafie C, Kleinfeld D. Topological basis for the robust distribution of blood to rodent neocortex. Proceedings of the National Academy of Sciences. 2010;107(28):12670–12675.

2. Adams MD, Winder AT, Blinder P, Drew PJ. The pial vasculature of the mouse develops according to a sensory-independent program. Scientific reports. 2018;8(1):1–12.

3. Binder N, El Amki M, Glueck C, Middleham W, Reuss AM, Bertolo A, et al. Leptomeningeal collaterals regulate reperfusion in ischemic stroke. Preprint at bioRxiv. 2023; p. 2023–02. doi:10.1101/2023.02.25.529915.

4. Voigt FF, Kirschenbaum D, Platonova E, Campbell RA, Kastli R, Schaettin M, et al. The mesoSPIM initiative: open-source light-sheet microscopes for imaging cleared tissue. Nature methods. 2019;16(11):1105–1108.

5. Renier N, Wu Z, Simon DJ, Yang J, Ariel P, Tessier-Lavigne M. iDISCO: a simple, rapid method to immunolabel large tissue samples for volume imaging. Cell. 2014;159(4):896–910.

6. Blinder P, Tsai PS, Kaufhold JP, Knutsen PM, Suhl H, Kleinfeld D. The cortical angiome: an interconnected vascular network with noncolumnar patterns of blood flow. Nature neuroscience. 2013;16(7):889–897.
