# Supplementary material for: The role of leptomeningeal collaterals in redistributing blood flow during stroke
Source: PLoS Comput Biol. 2023 Oct 23;19(10):e1011496. doi: 10.1371/journal.pcbi.1011496 (PMC10621965; doi:10.1371/journal.pcbi.1011496)
Supplement: S2 Appendix — References of S2 Appendix: [37, 39, 70, 95]. (PDF) [file pcbi.1011496.s015.pdf]

S2 Appendix. Blood flow model details.

Since Reynolds (Re) and Womersley (Wo) numbers are small in the microvasculature, i.e., Re$<$1 and Wo$<$1 in almost all vessels, Poiseuille's law was used to compute the blood flow rate q_{ij} in every edge e_{ij} based on the pressures p_i and p_j in the two adjacent vertices v_i and v_j, i.e.,

$$q_{ij} = \frac{\pi d_{ij}^4}{128 l_{ij}\ \mu_p\ \mu_{rel,ij}} (p_i - p_j). \tag{1}$$

Here, d_{ij} and l_{ij} are diameter and length of the vessel associated to e_{ij}, and $\mu_p = 0.0012\,\mathrm{Pa\,s}$ [1, 2] is the viscosity of blood plasma. The relative apparent viscosity $\mu_{rel,ij} = f(H_{t,ij}, d_{ij})$ depends on the tube haematocrit $H_{t,ij}$ and the vessel diameter, and is an empirical correction term [3] to account for the Fåhraeus-Lindqvist effect. For the current study, we assumed a constant $H_{t,ij} = 0.3$ [4] for all edges in the entire network.

As flow is conserved, the balance g_i for vertex v_i is

$$g_i = \sum_{v_k \in \mathcal{N}(v_i)} q_{ik} = 0, \tag{2}$$

where $\mathcal{N}(v_i)$ is the set of all neighbouring vertices to vertex v_i. By combining Eqs. (1) and (2) with appropriate boundary conditions, the pressure and flow fields can be computed for the entire network. The RBC velocity deviates from the bulk velocity due to the Fåhraeus effect, i.e.,

$$u_{ij} = \frac{4 q_{ij}}{d_{ij}^2 \pi} \frac{H_{d,ij}}{H_{t,ij}}. \tag{3}$$

Similarly to $\mu_{rel,ij}$, the ratio $H_{d,ij}/H_{t,ij}$, i.e., the ratio of discharge to tube haematocrit in vessel e_{ij}, was determined based on empirical functions [3].

References of S2 Appendix

1. Nader E, Skinner S, Romana M, Fort R, Lemonne N, Guillot N, et al. Blood rheology: key parameters, impact on blood flow, role in sickle cell disease and effects of exercise. Frontiers in physiology. 2019;10:1329.

2. Lorthois S, Cassot F, Lauwers F. Simulation study of brain blood flow regulation by intra-cortical arterioles in an anatomically accurate large human vascular network: Part I: Methodology and baseline flow. NeuroImage. 2011;54(2):1031–1042.

3. Pries AR, Neuhaus D, Gaehtgens P. Blood viscosity in tube flow: dependence on diameter and hematocrit. American Journal of Physiology-Heart and Circulatory Physiology. 1992;263(6):H1770–H1778.

4. Schmid F, Tsai PS, Kleinfeld D, Jenny P, Weber B. Depth-dependent flow and pressure characteristics in cortical microvascular networks. PLoS computational biology. 2017;13(2):e1005392.
