# Supplementary material for: The role of leptomeningeal collaterals in redistributing blood flow during stroke
Source: PLoS Comput Biol. 2023 Oct 23;19(10):e1011496. doi: 10.1371/journal.pcbi.1011496 (PMC10621965; doi:10.1371/journal.pcbi.1011496)
Supplement: S3 Appendix — References of S3 Appendix: [43, 70]. (PDF) [file pcbi.1011496.s016.pdf]

S3 Appendix. Inverse model details.

In the following we describe the inverse model [1] in detail. Please refer to S2 Appendix for the nomenclature related to the blood flow model which is used in this section. The inverse model was solved by iteratively minimizing a cost function $J^{(\nu)}$, where ν is the iteration step. The total cost function value is

$$J^{(\nu)} = J^{(\nu)}_{meas} + J^{(\nu)}_{range}, \tag{4}$$

where $J^{(\nu)}_{meas}$ and $J^{(\nu)}_{range}$ are the contributions to the total cost originating from the two different types of constraints, i.e.,

$$J^{(\nu)}_{meas} = \sum_{e_{ij} \in e_{meas}} \left(\frac{u^{(\nu)}_{ij} - u^{meas}_{ij}}{\sigma_{ij}} \right)^2 \tag{5}$$

and

$$J^{(\nu)}_{range} = \sum_{e_{ij} \in e_{range}} \begin{cases} \left(\frac{u^{(\nu)}_{ij} - u^{max}_{ij}}{\sigma_{ij}} \right)^2 & \text{, if } u^{(\nu)}_{ij} > u^{max}_{ij} \\ \left(\frac{u^{(\nu)}_{ij} - u^{min}_{ij}}{\sigma_{ij}} \right)^2 & \text{, if } u^{(\nu)}_{ij} < u^{min}_{ij} \\ 0 & \text{, else.} \end{cases} \tag{6}$$

Here, e_{meas} and e_{range} are the sets of edge ids with measurements of the respective type, and σ_{ij} is a weighting and normalisation term. The precise value of σ_{ij} primarily affects the convergence rates of the individual cost terms of $J^{(\nu)}$, and was chosen uniformly for all measurements. Minimizing Eq. (4) with a uniform σ_{ij} leads to solutions that minimise the absolute differences between simulated and target velocities. In contrast, choosing $\sigma_{ij} = u^{meas}_{ij} \; \forall \; e_{ij} \in (e_{meas}, e_{range})$ would lead to solutions with minimal relative differences. Note that $J^{(\nu)}_{range}$ consists of a sum of terms with zero cost in the range from u^{min}_{ij} to u^{max}_{ij}, and terms from $J^{(\nu)}_{meas}$ have only one single minima (Fig 2B). Similar to our previous publication [1], we searched for a minimum of the cost function by using a gradient-based approach, i.e.,

$$\alpha^{(\nu+1)}_{ij} = \alpha^{(\nu)}_{ij} - \gamma \frac{dJ^{(\nu)}}{d\alpha^{(\nu)}_{ij}}, \tag{7}$$

where $\alpha^{(\nu)}_{ij} = d^{(\nu)}_{ij} / d^{(0)}_{ij}$ is the relative diameter to the prior value $d^{(0)}_{ij}$. Furthermore, γ is a constant learning rate to control convergence and stability of the algorithm, i.e., $\gamma = 5$ for all scenarios considered in this study. The gradient $dJ^{(\nu)}/d\alpha^{(\nu)}_{ij}$ was calculated analytically with the adjoint method, i.e.,

$$\frac{dJ^{(\nu)}}{d\alpha^{(\nu)}_{ij}} = \sum_{l=1}^{N_v} \left(\lambda_l \frac{\partial g^{(\nu)}_l}{\partial \alpha^{(\nu)}_{ij}} \right) + \frac{\partial J^{(\nu)}}{\partial \alpha^{(\nu)}_{ij}}, \tag{8}$$

where λ_l is obtained from the adjoint equation, i.e., from

$$\sum_{l=1}^{N_v} \left(\frac{\partial g^{(\nu)}_l}{\partial p^{(\nu)}_i} \lambda_l \right) = -\frac{\partial J^{(\nu)}}{\partial p^{(\nu)}_i}. \tag{9}$$

Due to the sum rule, the two terms of the cost function can be treated separately while computing the partial derivatives, e.g.

$$\frac{\partial J^{(\nu)}}{\partial \alpha_{ij}^{(\nu)}} = \frac{\partial J_{meas}^{(\nu)}}{\partial \alpha_{ij}^{(\nu)}} + \frac{\partial J_{range}^{(\nu)}}{\partial \alpha_{ij}^{(\nu)}}. \tag{10}$$

The partial derivative $\partial J_{meas}^{(\nu)} / \partial \alpha_{ij}^{(\nu)}$ is

$$\frac{\partial J_{meas}^{(\nu)}}{\partial \alpha_{ij}^{(\nu)}} = \begin{cases} \frac{2(u_{ij}^{(\nu)} - u_{ij}^{meas})}{\sigma_{ij}^2} \cdot \frac{\partial u_{ij}^{(\nu)}}{\partial \alpha_{ij}^{(\nu)}} & \text{, if } e_{ij} \in e_{meas} \\ 0 & \text{, else,} \end{cases} \tag{11}$$

with

$$\frac{\partial u_{ij}^{(\nu)}}{\partial \alpha_{ij}^{(\nu)}} \approx \frac{4}{\pi {d_{ij}^{(0)}}^2} \frac{H_{d,ij}^{(\nu)}}{H_{t,ij}^{(\nu)}} \left(\frac{(p_i^{(\nu)} - p_j^{(\nu)})}{{\alpha_{ij}^{(\nu)}}^2} \cdot \frac{\partial T_{ij}^{(\nu)}}{\partial \alpha_{ij}^{(\nu)}} - \frac{2 q_{ij}^{(\nu)}}{{\alpha_{ij}^{(\nu)}}^3} \right) \tag{12}$$

and

$$\frac{\partial T_{ij}^{(\nu)}}{\partial \alpha_{ij}^{(\nu)}} \approx \frac{\pi {d_{ij}^{(0)}}^4}{32 l_{ij} \mu_p \mu_{rel,ij}^{(\nu)}} \cdot {\alpha_{ij}^{(\nu)}}^3. \tag{13}$$

In line with our previous work [1], Eqs. (12) and (13) assume $\partial \left(\frac{H_{d,ij}^{(\nu)}}{H_{t,ij}^{(\nu)}} \right) / \partial \alpha_{ij} \approx 0$ and $\partial \mu_{rel,ij}^{(\nu)} / \partial \alpha_{ij} \approx 0$. This simplification allows that no derivatives of the empirical laws [2] for Fåhraeus- and Fåhraeus-Linquist effects have to be computed, and only slightly reduces the overall convergence rate of the model. The partial derivative $\partial J_{meas}^{(\nu)} / \partial p_l^{(\nu)}$ is

$$\frac{\partial J_{meas}^{(\nu)}}{\partial p_l^{(\nu)}} = \sum_{e_{ij} \in e_{meas}} \left(\frac{2(u_{ij}^{(\nu)} - u_{ij}^{meas})}{\sigma_{ij}^2} \cdot \frac{\partial u_{ij}^{(\nu)}}{\partial p_l^{(\nu)}} \right) \tag{14}$$

with

$$\frac{\partial u_{ij}^{(\nu)}}{\partial p_l^{(\nu)}} = \frac{4 T_{ij}^{(\nu)}}{\pi {d_{ij}^{(\nu)}}^2} \frac{H_{d,ij}^{(\nu)}}{H_{t,ij}^{(\nu)}} \cdot \begin{cases} 1 & \text{, if } l = i \\ -1 & \text{, if } l = j \\ 0 & \text{, else,} \end{cases} \tag{15}$$

where the transmissibility $T_{ij}^{(\nu)}$ is defined as

$$T_{ij}^{(\nu)} = \frac{\pi {d_{ij}^{(\nu)}}^4}{128 l_{ij} \ \mu_p \ \mu_{rel,ij}^{(\nu)}}. \tag{16}$$

The computation of the partial derivatives $\partial J_{range}^{(\nu)} / \partial \alpha_{ij}^{(\nu)}$ and $\partial J_{range}^{(\nu)} / \partial p_l^{(\nu)}$ is very similar to Eqs. (11) and (14), and can be derived straightforwardly. Furthermore, since the derivatives $\partial g_l^{(\nu)} / \partial \alpha_{ij}^{(\nu)}$ and $\partial g_l^{(\nu)} / \partial p_i^{(\nu)}$ solely depend on the blood flow model and not on the precise formulation of the cost function, they are computed as in our previous work [1], i.e.,

$$\frac{\partial g_l^{(\nu)}}{\partial \alpha_{ij}^{(\nu)}} = \begin{cases} (p_i^{(\nu)} - p_j^{(\nu)}) \frac{\partial T_{ij}^{(\nu)}}{\partial \alpha_{ij}^{(\nu)}} & \text{, if } l = i \\ 0 & \text{, else} \end{cases} \tag{17}$$

and

$$\frac{\partial g_l^{(\nu)}}{\partial p_i^{(\nu)}} = \sum_{v_k \in \mathcal{N}(v_l)} T_{lk} (\delta_{li} - \delta_{ki}), \tag{18}$$

where δ_{ij} is the Kronecker delta.

References of S3 Appendix

1. Epp R, Schmid F, Weber B, Jenny P. Predicting vessel diameter changes to up-regulate biphasic blood flow during activation in realistic microvascular networks. Frontiers in physiology. 2020;11:1132.

2. Pries AR, Neuhaus D, Gaehtgens P. Blood viscosity in tube flow: dependence on diameter and hematocrit. American Journal of Physiology-Heart and Circulatory Physiology. 1992;263(6):H1770–H1778.
