# Supplementary material for: The role of leptomeningeal collaterals in redistributing blood flow during stroke
Source: PLoS Comput Biol. 2023 Oct 23;19(10):e1011496. doi: 10.1371/journal.pcbi.1011496 (PMC10621965; doi:10.1371/journal.pcbi.1011496)
Supplement: S4 Appendix — References of S4 Appendix: [21, 79, 81, 96, 97]. (PDF) [file pcbi.1011496.s017.pdf]

S4 Appendix. Vessel elasticity model details

Passive vascular diameter adaptations due to pressure changes after MCAo were taken into account by considering the elasticity of blood vessels. Please refer to S2 Appendix for the nomenclature related to the blood flow model used in this section. Here, we used a pressure-area relation derived from linear elastic theory [1–3] to compute the diameter d_{ij} based on a given average vessel pressure $p_{ij} = 0.5(p_i + p_j)$, i.e.,

$$d_{ij} = d_{ref,ij} + \frac{d_{ref,ij}^2(1-\nu^2)}{2E_{ij}h_{ij}}\left(p_{ij} - p_0\right). \tag{19}$$

Here, p_0 is a constant external pressure outside the blood vessel and ν, E_{ij} and h_{ij} are Poisson ratio, Young's modulus and wall thickness of the vessel material. In line with [3], we assumed $p_0 = 0\,\text{mmHg}$, $\nu = 0.5$, $h_{ij} = 0.1d_{ij}$ and values for E_{ij} as summarised in Table A in S4 Appendix). Furthermore, the reference diameters $d_{ref,ij}$ of all vessels were calculated based on the diameters and pressures at baseline conditions, i.e., after the network was tuned with the inverse model but before the MCAo was induced.

Table A in S4 Appendix. Young's modulus (E_{ij}) of different vessel types in the microvasculature. Table adapted from [3]. Original data from [4, 5].

Vessel type	E_{ij}
SAs & DAs ($d_{ij} \geq 20\,\mu\text{m}$)	$6.3 \times 10^5\text{Pa}$
SAs & DAs ($15\,\mu\text{m} \leq d_{ij} < 20\,\mu\text{m}$)	$2.6 \times 10^5\text{Pa}$
SAs & DAs ($d_{ij} < 15\,\mu\text{m}$)	$1.6 \times 10^5\text{Pa}$
Cs	$3.70 \times 10^5\text{Pa}$
AVs	$3.88 \times 10^5\text{Pa}$

Due to the two-way coupling of pressure and vessel diameter, the system was solved iteratively. In every iteration, blood flow rates and pressures were first calculated with the blood flow model (S2 Appendix Eqs. (1) and (2)). In a second step, the diameters were updated based on the current pressure distributions using the elasticity model (Eq. (19)). This procedure was repeated until the algorithm converged and the maximum diameter change compared to the previous iteration was less than 10^{-4} µm, which was generally the case after ≈ 4 iterations.

References of S4 Appendix

1. Sherwin S, Franke V, Peiró J, Parker K. One-dimensional modelling of a vascular network in space-time variables. Journal of engineering mathematics. 2003;47:217–250.

2. Padmos RM, Arrarte Terreros N, Józsa TI, Závodszky G, Marquering HA, Majoie CB, et al. Modelling collateral flow and thrombus permeability during acute ischaemic stroke. Journal of the Royal Society Interface. 2022;19(195):20220649.

3. Daher A, Payne S. A network-based model of dynamic cerebral autoregulation. Microvascular Research. 2023;147:104503.

4. Smaje L, Fraser P, Clough G. The distensibility of single capillaries and venules in the cat mesentery. Microvascular research. 1980;20(3):358–370.

5. Salotto AG, Muscarella LF, Melbin J, Li JKJ, Noordergraaf A. Pressure pulse transmission into vascular beds. Microvascular research. 1986;32(2):152–163.
