# Supplementary material for: The role of leptomeningeal collaterals in redistributing blood flow during stroke
Source: PLoS Comput Biol. 2023 Oct 23;19(10):e1011496. doi: 10.1371/journal.pcbi.1011496 (PMC10621965; doi:10.1371/journal.pcbi.1011496)
Supplement: S1 Table — For LMCs, diameter measurements are additionally given for the state after MCAo & LMC-dil. “x” is used if no velocity or diameter measurement was obtained in the vessel. The measurements are grouped into MCA and ACA sided SAs, and LMCs. Refer to S24, S25 and S26 Tables for measurements in other datasets. (PDF) [file pcbi.1011496.s018.pdf]

Supporting Tables.

S1 Table

#	Region	Diameter (Base) [µm]	Velocity (Base) [$mm\,s^{-1}$]	Diameter (MCAo & LMC-dil) [µm]
1	MCA	59.4	x	x
2	MCA	27.3	6.70	x
3	MCA	29.4	x	x
4	MCA	24.5	x	x
5	MCA	15.4	x	x
6	MCA	29.2	4.14	x
7	MCA	15.6	x	x
8	ACA	29.4	4.40	x
9	ACA	31.4	7.65	x
10	ACA	31.2	5.33	x
11	LMC	10.9	1.23	23.6
12	LMC	6.9	2.25	17.1
13	LMC	18.5	x	33.1
14	LMC	6.7	0.37	19.4
15	LMC	6.5	1.23	10.5
16	LMC	7.6	0.23	10.9
17	LMC	7.5	0.13	11.7
18	LMC	11.6	0.53	18.9
