# Supplementary material for: The role of leptomeningeal collaterals in redistributing blood flow during stroke
Source: PLoS Comput Biol. 2023 Oct 23;19(10):e1011496. doi: 10.1371/journal.pcbi.1011496 (PMC10621965; doi:10.1371/journal.pcbi.1011496)
Supplement: S2 Table — (PDF) [file pcbi.1011496.s019.pdf]

Supporting Tables.

S2 Table

	Nr. of edges [-]	Nr. of vertices [-]	Network size [mm^3]	Nr. of LMCs [-]	Nr. of MCA:ACA SAs [-]
C57BL/6_{I}	204116	146922	6.5 x 4.5 x 0.8	8	165:189
C57BL/6_{II}	208521	150208	5.8 x 4.7 x 0.8	4	251:92
BALB/c_{I}	174226	124160	5.9 x 4.6 x 0.8	x	142:68
BALB/c_{II}	192754	136775	5.7 x 5.5 x 0.8	x	176:87
