# Supplementary material for: The role of leptomeningeal collaterals in redistributing blood flow during stroke
Source: PLoS Comput Biol. 2023 Oct 23;19(10):e1011496. doi: 10.1371/journal.pcbi.1011496 (PMC10621965; doi:10.1371/journal.pcbi.1011496)
Supplement: S3 Table — Mean ± standard deviation of diameters after tuning were calculated by considering all edge segments of the respective vessel type. Furthermore, the ranges of reported mean literature values are given in the last row. References for literature values: ALength density [3, 65, 98, 99]; BVolume density [3, 45–48, 99, 100]; CSAs [17, 28, 101]; DDAs [3, 17, 28, 84, 88, 101–103]; ECs [3, 17, 46–48, 84, 88, 99, 101–103]; FAVs [3, 17, 88]. (PDF) [file pcbi.1011496.s020.pdf]

Supporting Tables.

S3 Table

	Length density [m/mm^3]	Volume density [mm^3/mm^3]	SAs: d [µm]	DAs: d [µm]	Cs: d [µm]	AVs: d [µm]
C57BL/6$_{\mathrm{I}}$	0.90	0.033	20.9 ± 6.2	10.5 ± 3.0	4.0 ± 0.2	12.0 ± 5.6
C57BL/6$_{\mathrm{II}}$	0.91	0.034	31.3 ± 10.7	10.4 ± 3.2	4.0 ± 0.1	12.0 ± 5.6
BALB/c$_{\mathrm{I}}$	0.79	0.027	27.9 ± 5.3	10.5 ± 3.2	4.0 ± 0.1	12.0 ± 5.6
BALB/c$_{\mathrm{II}}$	0.76	0.023	33.4 ± 8.9	10.4 ± 3.1	4.0 ± 0.1	12.0 ± 5.4
Literature	0.45 - 1.02[A]	0.01 - 0.036[B]	18.6 - 40.7[C]	8.6 - 25.6[D]	3.2 - 6.0[E]	9 - 34[F]
