# Supplementary material for: The role of leptomeningeal collaterals in redistributing blood flow during stroke
Source: PLoS Comput Biol. 2023 Oct 23;19(10):e1011496. doi: 10.1371/journal.pcbi.1011496 (PMC10621965; doi:10.1371/journal.pcbi.1011496)
Supplement: S5 Table — The relative change of mean pressures after MCAo in comparison to baseline was defined as ΔprelBase→MCAo=mean(pMCAo)-mean(pBase)mean(pBase),(2) where pBase and pMCAo are the pressure values at baseline and after MCAo, respectively. Analogously, the superscript MCAo → MCAo & LMC-dil denotes the relative change from MCAo to MCAo & LMC-dil. Refer to S13 Table for results after LMC/SA/DA-dil. (PDF) [file pcbi.1011496.s022.pdf]

Supporting Tables.

S5 Table

	$\Delta p_{rel}^{Base \rightarrow MCAo}$	$\Delta p_{rel}^{MCAo \rightarrow MCAo\&LMC-dil}$
**C57BL/6$_{\mathbf{I}}$:**		
MCA SAs	−60.0 %	+3.5 %
ACA SAs	−6.9 %	−4.2 %
**C57BL/6$_{\mathbf{II}}$:**		
MCA SAs	−74.3 %	+5.9 %
ACA SAs	−2.9 %	−5.0 %
