# Supplementary material for: The role of leptomeningeal collaterals in redistributing blood flow during stroke
Source: PLoS Comput Biol. 2023 Oct 23;19(10):e1011496. doi: 10.1371/journal.pcbi.1011496 (PMC10621965; doi:10.1371/journal.pcbi.1011496)
Supplement: S6 Table — The relative change of mean flow rate after MCAo in comparison to baseline was defined as ΔqrelBase→MCAo=mean(qMCAo)-mean(qBase)mean(qBase),(3) where qBase and qMCAo are the flow rates at baseline and after MCAo. Analogously, the superscripts MCAo → MCAo & LMC-dil and Base → MCAo & LMC-dil denote relative changes from MCAo to MCAo & LMC-dil and from baseline to MCAo & LMC-dil, respectively. Refer to S14 Table for results after LMC/SA/DA-dil. (PDF) [file pcbi.1011496.s023.pdf]

Supporting Tables.

S6 Table

	$\Delta q_{rel}^{Base \rightarrow MCAo}$	$\Delta q_{rel}^{MCAo \rightarrow MCAo\&LMC-dil}$	$\Delta q_{rel}^{Base \rightarrow MCAo\&LMC-dil}$
**C57BL/6$_{\mathbf{I}}$:**			
MCA SAs, overall	−95.0 %	+22.3 %	−93.9 %
MCA SAs, *path to LMCs*	−95.5 %	+18.5 %	−94.6 %
MCA SAs, *others*	−94.0 %	+28.1 %	−92.3 %
ACA SAs, overall	+4.2 %	+1.2 %	+5.5 %
ACA SAs, *path to LMCs*	+15.0 %	+7.1 %	+23.1 %
ACA SAs, *others*	−2.7 %	−3.3 %	−5.9 %
LMCs	+758.5 %	+91.0 %	+1539.7 %
**C57BL/6$_{\mathbf{II}}$:**			
MCA SAs, overall	−97.8 %	+37.0 %	−97.0 %
MCA SAs, *path to LMCs*	−98.3 %	+18.9 %	−98.0 %
MCA SAs, *others*	−97.0 %	+52.1 %	−95.4 %
ACA SAs, overall	+1.4 %	+1.2 %	+2.6 %
ACA SAs, *path to LMCs*	+3.3 %	+8.8 %	+12.4 %
ACA SAs, *others*	−0.4 %	−6.0 %	−6.4 %
LMCs	+1050.7 %	+569.6 %	+7605.2 %
