# Supplementary material for: The role of leptomeningeal collaterals in redistributing blood flow during stroke
Source: PLoS Comput Biol. 2023 Oct 23;19(10):e1011496. doi: 10.1371/journal.pcbi.1011496 (PMC10621965; doi:10.1371/journal.pcbi.1011496)
Supplement: S7 Table — The relative change of integral flow rate after MCAo in comparison to baseline was defined as ΔQrelBase→MCAo=sum(qMCAo)-sum(qBase)sum(qBase),(4) where qBase and qMCAo are the flow rates at baseline and after MCAo. Analogously, the superscripts MCAo → MCAo & LMC-dil and Base → MCAo & LMC-dil denote relative changes from MCAo to MCAo & LMC-dil and from baseline to MCAo & LMC-dil, respectively. Refer to S15 Table for results after LMC/SA/DA-dil. (PDF) [file pcbi.1011496.s024.pdf]

Supporting Tables.

S7 Table

	$\Delta Q_{rel}^{Base \to MCAo}$	$\Delta Q_{rel}^{MCAo \to MCAo\&LMC-dil}$	$\Delta Q_{rel}^{Base \to MCAo\&LMC-dil}$
**C57BL/6$_{\mathbf{I}}$:**			
MCA DAs, overall	−92.6 %	+38.9 %	−89.7 %
MCA DAs, $r < 250\mu m$	−88.2 %	+63.9 %	−80.6 %
ACA DAs, overall	−5.7 %	−5.0 %	−10.4 %
ACA DAs, $r < 250\mu m$	−19.1 %	−33.4 %	−46.1 %
**C57BL/6$_{\mathbf{II}}$:**			
MCA DAs, overall	−96.8 %	+53.8 %	−95.1 %
MCA DAs, $r < 250\mu m$	−97.4 %	+162.9 %	−93.1 %
ACA DAs, overall	−0.6 %	−7.4 %	−8.0 %
ACA DAs, $r < 250\mu m$	−0.5 %	−11.8 %	−12.2 %
