# Supplementary material for: The role of leptomeningeal collaterals in redistributing blood flow during stroke
Source: PLoS Comput Biol. 2023 Oct 23;19(10):e1011496. doi: 10.1371/journal.pcbi.1011496 (PMC10621965; doi:10.1371/journal.pcbi.1011496)
Supplement: S8 Table — Refer to the caption of S7 Table for the definitions of ΔQrelBase→MCAo, ΔQrelMCAo→MCAo&LMC-dil and ΔQrelBase→MCAo&LMC-dil. Refer to S16 Table for results after LMC/SA/DA-dil. (PDF) [file pcbi.1011496.s025.pdf]

Supporting Tables.

S8 Table

	$\Delta Q_{rel}^{Base \to MCAo}$	$\Delta Q_{rel}^{MCAo \to MCAo\&LMC-dil}$	$\Delta Q_{rel}^{Base \to MCAo\&LMC-dil}$
**C57BL/6$_{\mathbf{I}}$:**			
MCA Cs, overall	−91.7 %	+25.5 %	−89.6 %
MCA Cs, $r < 250\mu m$	−84.8 %	+32.3 %	−79.9 %
ACA Cs, overall	−14.7 %	−5.2 %	−19.2 %
ACA Cs, $r < 250\mu m$	−51.9 %	−23.0 %	−63.0 %
**C57BL/6$_{\mathbf{II}}$:**			
MCA Cs, overall	−94.6 %	+26.6 %	−93.1 %
MCA Cs, $r < 250\mu m$	−89.9 %	+22.1 %	−87.7 %
ACA Cs, overall	−11.4 %	−6.8 %	−17.4 %
ACA Cs, $r < 250\mu m$	−17.3 %	−10.4 %	−26.0 %
