# Supplementary material for: The role of leptomeningeal collaterals in redistributing blood flow during stroke
Source: PLoS Comput Biol. 2023 Oct 23;19(10):e1011496. doi: 10.1371/journal.pcbi.1011496 (PMC10621965; doi:10.1371/journal.pcbi.1011496)
Supplement: S9 Table — 〈…〉 is used to refer to average values of all four datasets. The results are consistent with the bars in Fig 5B. Refer to S18 Table for results after LMC/SA/DA-dil. (PDF) [file pcbi.1011496.s026.pdf]

Supporting Tables.

S9 Table

	$\left\langle \Delta q_{rel}^{Base \rightarrow MCAo} \right\rangle$	$\left\langle \Delta q_{rel}^{MCAo \rightarrow MCAo\&LMC-dil} \right\rangle$	$\left\langle \Delta q_{rel}^{Base \rightarrow MCAo\&LMC-dil} \right\rangle$
MCA SAs on paths to LMCs:			
100 % LMC	−96.1 %	+78.8 %	−92.8 %
50 % LMC	−96.2 %	+51.9 %	−94.3 %
0 % LMC	−96.5 %	x	x
Other MCA SAs:			
100 % LMC	−93.9 %	+73.1 %	−89.3 %
50 % LMC	−94.4 %	+50.4 %	−91.4 %
0 % LMC	−95.6 %	x	x
ACA SAs on paths to LMCs:			
100 % LMC	+8.2 %	+11.5 %	+20.5 %
50 % LMC	+6.5 %	+7.5 %	+14.5 %
0 % LMC	+2.4 %	x	x
Other ACA SAs:			
100 % LMC	−2.3 %	−6.4 %	−8.6 %
50 % LMC	−1.4 %	−3.8 %	−5.1 %
0 % LMC	+0.1 %	x	x
