# Supplementary material for: The role of leptomeningeal collaterals in redistributing blood flow during stroke
Source: PLoS Comput Biol. 2023 Oct 23;19(10):e1011496. doi: 10.1371/journal.pcbi.1011496 (PMC10621965; doi:10.1371/journal.pcbi.1011496)
Supplement: S10 Table — 〈…〉 is used to refer to average values of all four datasets. Refer to S17 Table for results after LMC/SA/DA-dil. (PDF) [file pcbi.1011496.s027.pdf]

Supporting Tables.

S10 Table

	$\left\langle \Delta p_{rel}^{Base \rightarrow MCAo} \right\rangle$	$\left\langle \Delta p_{rel}^{MCAo \rightarrow MCAo\&LMC-dil} \right\rangle$
MCA SAs:		
100 % LMC	−70.0 %	+16.4 %
50 % LMC	−70.5 %	+10.9 %
0 % LMC	−71.7 %	x
ACA SAs:		
100 % LMC	−4.2 %	−5.8 %
50 % LMC	−3.3 %	−3.6 %
0 % LMC	−1.5 %	x
