# Supplementary material for: The role of leptomeningeal collaterals in redistributing blood flow during stroke
Source: PLoS Comput Biol. 2023 Oct 23;19(10):e1011496. doi: 10.1371/journal.pcbi.1011496 (PMC10621965; doi:10.1371/journal.pcbi.1011496)
Supplement: S11 Table — 〈…〉 is used to refer to average values computed over all four datasets. The results are consistent with the bars in Fig 5C and 5D. Refer to S19 Table for results after LMC/SA/DA-dil. (PDF) [file pcbi.1011496.s028.pdf]

# Supporting Tables.

**S11 Table**

| | $\left\langle\Delta Q_{rel}^{Base\rightarrow MCAo}\right\rangle$ | $\left\langle\Delta Q_{rel}^{MCAo\rightarrow MCAo\&LMC-dil}\right\rangle$ | $\left\langle\Delta Q_{rel}^{Base\rightarrow MCAo\&LMC-dil}\right\rangle$ |
|---|---|---|---|
| *MCA DAs, overall:* | | | |
| 100 % LMC | −93.4 % | +82.9 % | −87.9 % |
| 50 % LMC | −94.1 % | +55.7 % | −90.6 % |
| 0 % LMC | −95.5 % | x | x |
| *MCA DAs, $r < 250$ μm:* | | | |
| 100 % LMC | −92.1 % | +205.7 % | −82.5 % |
| 50 % LMC | −93.1 % | +143.3 % | −87.1 % |
| 0 % LMC | −95.4 % | x | x |
| *ACA DAs, overall:* | | | |
| 100 % LMC | −2.7 % | −8.3 % | −10.8 % |
| 50 % LMC | −1.6 % | −5.0 % | −6.5 % |
| 0 % LMC | +0.8 % | x | x |
| *ACA DAs, $r < 250$ μm:* | | | |
| 100 % LMC | −4.4 % | −19.3 % | −22.1 % |
| 50 % LMC | −0.9 % | −10.3 % | −10.9 % |
| 0 % LMC | +7.9 % | x | x |
