# Supplementary material for: The role of leptomeningeal collaterals in redistributing blood flow during stroke
Source: PLoS Comput Biol. 2023 Oct 23;19(10):e1011496. doi: 10.1371/journal.pcbi.1011496 (PMC10621965; doi:10.1371/journal.pcbi.1011496)
Supplement: S12 Table — 〈…〉 is used to refer to average values computed over all four datasets. The results are consistent with the bars in Fig 5E and 5F. Refer to S20 Table for results after LMC/SA/DA-dil. (PDF) [file pcbi.1011496.s029.pdf]

Supporting Tables.

S12 Table

	$\left\langle \Delta Q_{rel}^{Base \rightarrow MCAo} \right\rangle$	$\left\langle \Delta Q_{rel}^{MCAo \rightarrow MCAo\&LMC-dil} \right\rangle$	$\left\langle \Delta Q_{rel}^{Base \rightarrow MCAo\&LMC-dil} \right\rangle$
MCA Cs, overall:			
100 % LMC	−89.4 %	+38.9 %	−85.1 %
50 % LMC	−89.9 %	+24.3 %	−87.2 %
0 % LMC	−90.9 %	x	x
MCA Cs, $r < 250\,\mu m$:			
100 % LMC	−79.4 %	+27.3 %	−74.2 %
50 % LMC	−79.9 %	+16.5 %	−76.7 %
0 % LMC	−81.3 %	x	x
ACA Cs, overall:			
100 % LMC	−10.3 %	−7.9 %	−17.4 %
50 % LMC	−9.3 %	−4.8 %	−13.6 %
0 % LMC	−7.1 %	x	x
ACA Cs, $r < 250\,\mu m$:			
100 % LMC	−24.0 %	−14.8 %	−34.5 %
50 % LMC	−22.0 %	−8.5 %	−28.3 %
0 % LMC	−17.7 %	x	x
