# Supplementary material for: The role of leptomeningeal collaterals in redistributing blood flow during stroke
Source: PLoS Comput Biol. 2023 Oct 23;19(10):e1011496. doi: 10.1371/journal.pcbi.1011496 (PMC10621965; doi:10.1371/journal.pcbi.1011496)
Supplement: S13 Table — (PDF) [file pcbi.1011496.s030.pdf]

Supporting Tables.

S13 Table

	$\Delta p_{rel}^{MCAo \rightarrow MCAo\&LMC/SA/DA-dil}$	$\Delta p_{rel}^{MCAo\&LMC-dil \rightarrow MCAo\&LMC/SA/DA-dil}$
**C57BL/6$_{\mathbf{I}}$:**		
MCA SAs	+4.8 %	+1.2 %
ACA SAs	−3.5 %	+0.8 %
**C57BL/6$_{\mathbf{II}}$:**		
MCA SAs	+5.4 %	−0.5 %
ACA SAs	−2.7 %	+2.4 %
