# Supplementary material for: The role of leptomeningeal collaterals in redistributing blood flow during stroke
Source: PLoS Comput Biol. 2023 Oct 23;19(10):e1011496. doi: 10.1371/journal.pcbi.1011496 (PMC10621965; doi:10.1371/journal.pcbi.1011496)
Supplement: S14 Table — (PDF) [file pcbi.1011496.s031.pdf]

Supporting Tables.

S14 Table

	$\Delta q_{rel}^{Base \to MCAo\&LMC/SA/DA-dil}$	$\Delta q_{rel}^{MCAo \to MCAo\&LMC/SA/DA-dil}$
**C57BL/6$_{\mathbf{I}}$:**		
MCA SAs, overall	−92.7 %	+46.2 %
MCA SAs, *path to LMCs*	−93.8 %	+36.3 %
MCA SAs, *others*	−90.2 %	+61.7 %
ACA SAs, overall	+21.8 %	+16.8 %
ACA SAs, *path to LMCs*	+49.4 %	+30 %
ACA SAs, *others*	+3.8 %	+6.7 %
LMCs	+2086.1 %	+154.7 %
**C57BL/6$_{\mathbf{II}}$:**		
MCA SAs, overall	−96.6 %	+52.7 %
MCA SAs, *path to LMCs*	−97.8 %	+29.1 %
MCA SAs, *others*	−94.8 %	+72.3 %
ACA SAs, overall	+20.4 %	+18.8 %
ACA SAs, *path to LMCs*	+30.4 %	+26.2 %
ACA SAs, *others*	+11.3 %	+11.8 %
LMCs	+8237.6 %	+624.6 %
