# Supplementary material for: The role of leptomeningeal collaterals in redistributing blood flow during stroke
Source: PLoS Comput Biol. 2023 Oct 23;19(10):e1011496. doi: 10.1371/journal.pcbi.1011496 (PMC10621965; doi:10.1371/journal.pcbi.1011496)
Supplement: S15 Table — (PDF) [file pcbi.1011496.s032.pdf]

Supporting Tables.

S15 Table

	$\Delta Q_{rel}^{Base \rightarrow MCAo\&LMC/SA/DA-dil}$	$\Delta Q_{rel}^{MCAo \rightarrow MCAo\&LMC/SA/DA-dil}$
**C57BL/6$_{\mathbf{I}}$:**		
MCA DAs, overall	−87.7 %	+67.1 %
MCA DAs, $r < 250\mu m$	−76.3 %	+99.9 %
ACA DAs, overall	−2.3 %	+3.6 %
ACA DAs, $r < 250\mu m$	−32.9 %	−17.1 %
**C57BL/6$_{\mathbf{II}}$:**		
MCA DAs, overall	−94.7 %	+66.2 %
MCA DAs, $r < 250\mu m$	−93.3 %	+156.5 %
ACA DAs, overall	+4.7 %	+5.3 %
ACA DAs, $r < 250\mu m$	−1.7 %	−1.3 %
