# Supplementary material for: The role of leptomeningeal collaterals in redistributing blood flow during stroke
Source: PLoS Comput Biol. 2023 Oct 23;19(10):e1011496. doi: 10.1371/journal.pcbi.1011496 (PMC10621965; doi:10.1371/journal.pcbi.1011496)
Supplement: S16 Table — (PDF) [file pcbi.1011496.s033.pdf]

Supporting Tables.

S16 Table

	$\Delta Q_{rel}^{Base \rightarrow MCAo\&LMC/SA/DA-dil}$	$\Delta Q_{rel}^{MCAo \rightarrow MCAo\&LMC/SA/DA-dil}$
**C57BL/6$_{\mathbf{I}}$:**		
MCA Cs, overall	−87.6 %	+48.4 %
MCA Cs, $r < 250 \mu m$	−75.8 %	+59.4 %
ACA Cs, overall	−12.3 %	+2.8 %
ACA Cs, $r < 250 \mu m$	−56.0 %	−8.5 %
**C57BL/6$_{\mathbf{II}}$:**		
MCA Cs, overall	−92.6 %	+36.9 %
MCA Cs, $r < 250 \mu m$	−86.7 %	+32.0 %
ACA Cs, overall	−8.4 %	+3.4 %
ACA Cs, $r < 250 \mu m$	−18.4 %	−1.3 %
