# Supplementary material for: The role of leptomeningeal collaterals in redistributing blood flow during stroke
Source: PLoS Comput Biol. 2023 Oct 23;19(10):e1011496. doi: 10.1371/journal.pcbi.1011496 (PMC10621965; doi:10.1371/journal.pcbi.1011496)
Supplement: S17 Table — 〈…〉 is used to refer to average values computed over all four datasets. (PDF) [file pcbi.1011496.s034.pdf]

Supporting Tables.

S17 Table

	$\left\langle \Delta p_{rel}^{MCAo \rightarrow MCAo\&LMC/SA/DA-dil} \right\rangle$
MCA SAs:	
100 % LMC	+17.0 %
50 % LMC	+11.5 %
0 % LMC	−0.7 %
ACA SAs:	
100 % LMC	−5.4 %
50 % LMC	−3.1 %
0 % LMC	+1.0 %
