# Supplementary material for: The role of leptomeningeal collaterals in redistributing blood flow during stroke
Source: PLoS Comput Biol. 2023 Oct 23;19(10):e1011496. doi: 10.1371/journal.pcbi.1011496 (PMC10621965; doi:10.1371/journal.pcbi.1011496)
Supplement: S18 Table — 〈…〉 is used to refer to average values computed over all four datasets. (PDF) [file pcbi.1011496.s035.pdf]

Supporting Tables.

S18 Table

	$\left\langle \Delta q_{rel}^{Base \to MCAo\&LMC/SA/DA-dil} \right\rangle$	$\left\langle \Delta q_{rel}^{MCAo \to MCAo\&LMC/SA/DA-dil} \right\rangle$
MCA SAs on paths to LMCs:		
100 % LMC	−91.7 %	+105.1 %
50 % LMC	−93.4 %	+77.0 %
0 % LMC	−96.3 %	+4.6 %
Other MCA SAs:		
100 % LMC	−87.4 %	+103.6 %
50 % LMC	−89.8 %	+77.7 %
0 % LMC	−95.1 %	+11.1 %
ACA SAs on paths to LMCs:		
100 % LMC	+37.8 %	+27.3 %
50 % LMC	+30.6 %	+22.7 %
0 % LMC	+15.1 %	+12.3 %
Other ACA SAs:		
100 % LMC	+0.4 %	+2.7 %
50 % LMC	+4.3 %	+5.7 %
0 % LMC	+10.5 %	+10.4 %
