# Supplementary material for: The role of leptomeningeal collaterals in redistributing blood flow during stroke
Source: PLoS Comput Biol. 2023 Oct 23;19(10):e1011496. doi: 10.1371/journal.pcbi.1011496 (PMC10621965; doi:10.1371/journal.pcbi.1011496)
Supplement: S19 Table — 〈…〉 is used to refer to average values computed over all four datasets. (PDF) [file pcbi.1011496.s036.pdf]

Supporting Tables.

S19 Table

	$\left\langle \Delta Q_{rel}^{Base \rightarrow MCAo\&LMC/SA/DA-dil} \right\rangle$	$\left\langle \Delta Q_{rel}^{MCAo \rightarrow MCAo\&LMC/SA/DA-dil} \right\rangle$
MCA DAs, overall:		
100 % LMC	−86.4 %	+105.6 %
50 % LMC	−89.3 %	+76.8 %
0 % LMC	−95.0 %	+9.4 %
MCA DAs, $r < 250$ µm*:*		
100 % LMC	−80.8 %	+216.9 %
50 % LMC	−85.6 %	+156.1 %
0 % LMC	−94.4 %	+21.3 %
ACA DAs, overall:		
100 % LMC	−2.9 %	−0.2 %
50 % LMC	+1.6 %	+3.2 %
0 % LMC	+10.2 %	+9.4 %
ACA DAs, $r < 250$ µm*:*		
100 % LMC	−11.1 %	−7.6 %
50 % LMC	+0.3 %	+1.1 %
0 % LMC	+22.5 %	+13.1 %
