# Supplementary material for: The role of leptomeningeal collaterals in redistributing blood flow during stroke
Source: PLoS Comput Biol. 2023 Oct 23;19(10):e1011496. doi: 10.1371/journal.pcbi.1011496 (PMC10621965; doi:10.1371/journal.pcbi.1011496)
Supplement: S20 Table — 〈…〉 is used to refer to average values computed over all four datasets. (PDF) [file pcbi.1011496.s037.pdf]

Supporting Tables.

S20 Table

	$\left\langle \Delta Q_{rel}^{Base \rightarrow MCAo\&LMC/SA/DA-dil} \right\rangle$	$\left\langle \Delta Q_{rel}^{MCAo \rightarrow MCAo\&LMC/SA/DA-dil} \right\rangle$
MCA Cs, overall:		
100 % LMC	−83.7 %	+53.6 %
50 % LMC	−86.0 %	+37.6 %
0 % LMC	−90.3 %	+7.4 %
MCA Cs, $r < 250\,\mu m$*:*		
100 % LMC	−72.5 %	+38.3 %
50 % LMC	−75.0 %	+27.8 %
0 % LMC	−79.9 %	+9.9 %
ACA Cs, overall:		
100 % LMC	−11.6 %	−1.4 %
50 % LMC	−7.6 %	+1.9 %
0 % LMC	0.0 %	+7.7 %
ACA Cs, $r < 250\,\mu m$*:*		
100 % LMC	−27.7 %	−5.3 %
50 % LMC	−21.6 %	+0.6 %
0 % LMC	−9.5 %	+10.5 %
