# Supplementary material for: The role of leptomeningeal collaterals in redistributing blood flow during stroke
Source: PLoS Comput Biol. 2023 Oct 23;19(10):e1011496. doi: 10.1371/journal.pcbi.1011496 (PMC10621965; doi:10.1371/journal.pcbi.1011496)
Supplement: S21 Table — (PDF) [file pcbi.1011496.s038.pdf]

Supporting Tables.

S21 Table

	$\Delta q_{rel}^{Base \to MCAo}\|_{elastic}$	$\Delta q_{rel}^{Base \to MCAo}\|_{rigid}$
**C57BL/6$_{\mathbf{I}}$:**		
MCA SAs, overall	−95.0 %	−94.9 %
MCA SAs, *path to LMCs*	−95.5 %	−95.5 %
MCA SAs, *others*	−94.0 %	−93.9 %
ACA SAs, overall	+4.2 %	+4.6 %
ACA SAs, *path to LMCs*	+15.0 %	+15.9 %
ACA SAs, *others*	−2.7 %	−2.8 %
LMCs	+758.5 %	+795.8 %
**C57BL/6$_{\mathbf{II}}$:**		
MCA SAs, overall	−97.8 %	−97.8 %
MCA SAs, *path to LMCs*	−98.3 %	−98.4 %
MCA SAs, *others*	−97.0 %	−96.9 %
ACA SAs, overall	+1.4 %	+1.7 %
ACA SAs, *path to LMCs*	+3.3 %	+3.9 %
ACA SAs, *others*	−0.4 %	−0.4 %
LMCs	+1050.7 %	+1215.0 %
