# Supplementary material for: The role of leptomeningeal collaterals in redistributing blood flow during stroke
Source: PLoS Comput Biol. 2023 Oct 23;19(10):e1011496. doi: 10.1371/journal.pcbi.1011496 (PMC10621965; doi:10.1371/journal.pcbi.1011496)
Supplement: S22 Table — (PDF) [file pcbi.1011496.s039.pdf]

Supporting Tables.

S22 Table

	$\Delta Q_{rel}^{Base \rightarrow MCAo}|_{elastic}$	$\Delta Q_{rel}^{Base \rightarrow MCAo}|_{rigid}$
**C57BL/6$_{\mathbf{I}}$:**		
MCA DAs, overall	−92.6 %	−92.5 %
MCA DAs, $r < 250\mu m$	−88.2 %	−88.0 %
ACA DAs, overall	−5.7 %	−5.8 %
ACA DAs, $r < 250\mu m$	−19.1 %	−18.9 %
**C57BL/6$_{\mathbf{II}}$:**		
MCA DAs, overall	−96.8 %	−96.7 %
MCA DAs, $r < 250\mu m$	−97.4 %	−97.3 %
ACA DAs, overall	−0.6 %	−0.5 %
ACA DAs, $r < 250\mu m$	−0.5 %	−0.3 %
