# Supplementary material for: The role of leptomeningeal collaterals in redistributing blood flow during stroke
Source: PLoS Comput Biol. 2023 Oct 23;19(10):e1011496. doi: 10.1371/journal.pcbi.1011496 (PMC10621965; doi:10.1371/journal.pcbi.1011496)
Supplement: S23 Table — (PDF) [file pcbi.1011496.s040.pdf]

Supporting Tables.

S23 Table

	$\Delta Q_{rel}^{Base \to MCAo}\|_{elastic}$	$\Delta Q_{rel}^{Base \to MCAo}\|_{rigid}$
**C57BL/6$_{\mathbf{I}}$:**		
MCA Cs, overall	$-91.7\,\%$	$-91.5\,\%$
MCA Cs, $r < 250\mu m$	$-84.8\,\%$	$-84.6\,\%$
ACA Cs, overall	$-14.7\,\%$	$-14.8\,\%$
ACA Cs, $r < 250\mu m$	$-51.9\,\%$	$-51.8\,\%$
**C57BL/6$_{\mathbf{II}}$:**		
MCA Cs, overall	$-94.6\,\%$	$-94.4\,\%$
MCA Cs, $r < 250\mu m$	$-89.9\,\%$	$-89.7\,\%$
ACA Cs, overall	$-11.4\,\%$	$-11.4\,\%$
ACA Cs, $r < 250\mu m$	$-17.3\,\%$	$-17.3\,\%$
