# Supplementary material for: The role of leptomeningeal collaterals in redistributing blood flow during stroke
Source: PLoS Comput Biol. 2023 Oct 23;19(10):e1011496. doi: 10.1371/journal.pcbi.1011496 (PMC10621965; doi:10.1371/journal.pcbi.1011496)
Supplement: S24 Table — “x” is used if no velocity or diameter measurement was obtained in the vessel. The measurements are grouped into MCA and ACA sided SAs. (PDF) [file pcbi.1011496.s041.pdf]

Supporting Tables.

S24 Table

#	Region	Diameter (Base) [µm]	Velocity (Base) [$mm\,s^{-1}$]
1	MCA	51.1	x
2	MCA	26.4	x
3	MCA	30.1	x
4	MCA	34.6	x
5	MCA	15.3	5.79
6	MCA	24.5	x
7	MCA	35.0	x
8	ACA	35.5	3.59
9	ACA	30.1	6.33
10	ACA	40.1	4.27
