# Supplementary material for: The role of leptomeningeal collaterals in redistributing blood flow during stroke
Source: PLoS Comput Biol. 2023 Oct 23;19(10):e1011496. doi: 10.1371/journal.pcbi.1011496 (PMC10621965; doi:10.1371/journal.pcbi.1011496)
Supplement: S25 Table — The measurements are grouped into MCA and ACA sided SAs. (PDF) [file pcbi.1011496.s042.pdf]

Supporting Tables.

S25 Table

#	Region	Diameter (Base) [µm]
1	MCA	39.5
2	MCA	53.4
3	MCA	36.9
4	MCA	54.8
5	MCA	45.6
6	MCA	54.5
7	MCA	48.3
8	MCA	25.9
9	MCA	22.2
10	MCA	28.6
11	MCA	21.6
12	MCA	30.8
13	MCA	32.1
14	MCA	25.0
15	MCA	25.9
16	ACA	27.3
17	ACA	17.5
18	ACA	26.3
19	MCA	21.2
