# Supplementary material for: The role of leptomeningeal collaterals in redistributing blood flow during stroke
Source: PLoS Comput Biol. 2023 Oct 23;19(10):e1011496. doi: 10.1371/journal.pcbi.1011496 (PMC10621965; doi:10.1371/journal.pcbi.1011496)
Supplement: S26 Table — The measurements are grouped into MCA and ACA sided SAs. (PDF) [file pcbi.1011496.s043.pdf]

Supporting Tables.

S26 Table

#	Region	Diameter (Base) [µm]
1	MCA	26.8
2	MCA	48.7
3	MCA	29.7
4	MCA	35.8
5	MCA	59.6
6	MCA	42.3
7	MCA	62.2
8	MCA	46.4
9	ACA	22.9
10	ACA	44.0
11	ACA	29.9
12	ACA	46.0
13	ACA	35.9
14	ACA	34.2
