# Supplementary material for: The role of leptomeningeal collaterals in redistributing blood flow during stroke
Source: PLoS Comput Biol. 2023 Oct 23;19(10):e1011496. doi: 10.1371/journal.pcbi.1011496 (PMC10621965; doi:10.1371/journal.pcbi.1011496)
Supplement: S27 Table — The average blood flow per surface area and tissue volume were determined based on the total blood flow that enters the network and the estimated surface area of the network, which was calculated from the Voronoi polygons shown in Fig A panel B in S1 Appendix. Based on the average blood flow per tissue volume, cerebral blood flow per mass was computed assuming a tissue density of 1046 kg/m3 [106]. References for CBF literature values: ACBF [107–111]. (PDF) [file pcbi.1011496.s044.pdf]

Supporting Tables.

S27 Table

	Avg. blood flow per area [$nL\,s^{-1}\,mm^{-2}$]	Avg. blood flow per tissue volume [$nL\,s^{-1}\,mm^{-3}$]	Avg. CBF [$ml\,min^{-1}\,g^{-1}$]
C57BL/6_{I}	12.2	15.1	0.87
C57BL/6_{II}	21.2	26.3	1.51
BALB/c_{I}	13.7	16.9	0.97
BALB/c_{II}	17.7	21.9	1.26
Literature	x	x	0.95 - 1.75[A]
