# Supplementary material for: The role of leptomeningeal collaterals in redistributing blood flow during stroke
Source: PLoS Comput Biol. 2023 Oct 23;19(10):e1011496. doi: 10.1371/journal.pcbi.1011496 (PMC10621965; doi:10.1371/journal.pcbi.1011496)
Supplement: S28 Table — Mean ± standard deviation are given. Capillaries at the border of the networks, i.e., with a distance >200 μm to any DA edge, were excluded from the analysis. The average values for DAs and AVs refer to the segments of the penetrating trees closest to the cortical surface, i.e., the DA and AV root edges. The ranges of reported mean literature values are given in the last row. References for literature values: ADAs [17, 37, 71, 92]; BCs [17, 37, 71, 88, 89, 91]; CAVs [17, 71]. (PDF) [file pcbi.1011496.s045.pdf]

Supporting Tables.

S28 Table

	DAs: q_{rbc} [nl/s]	Cs: q_{rbc} [pl/s]	AVs: q_{rbc} [nl/s]
$C57BL/6_{I}$	0.37 ± 0.49	2.0 ± 6.2	0.13 ± 0.20
$C57BL/6_{II}$	0.65 ± 0.53	3.6 ± 7.8	0.23 ± 0.26
$BALB/c_{I}$	0.63 ± 0.52	3.5 ± 6.1	0.21 ± 0.26
$BALB/c_{II}$	0.81 ± 0.62	5.0 ± 8.7	0.28 ± 0.29
Literature	0.8 - 2.3[A]	1.7 - 10[B]	0.2 - 1[C]
