# Supplementary material for: The role of leptomeningeal collaterals in redistributing blood flow during stroke
Source: PLoS Comput Biol. 2023 Oct 23;19(10):e1011496. doi: 10.1371/journal.pcbi.1011496 (PMC10621965; doi:10.1371/journal.pcbi.1011496)
Supplement: S29 Table — The wall shear stress in each vessel was estimated based on the flow rate qij, the diameter dij and the effective viscosity μeff,ij = μp μrel, ij (please refer to S2 Appendix for the nomenclature) by assuming a parabolic velocity profile, i.e., WSSij=32qijμeff,ijπdij3.(5) Mean ± standard deviation are given for all vessel types. Capillaries at the border of the networks, i.e., with a distance >200 μm to any DA edge, were excluded from the analysis. The average values for DAs and AVs refer to the segments of the penetrating trees closest to the cortical surface, i.e., the DA and AV root edges. The ranges of reported mean literature values obtained for the mesentery [112–114] and from a simulation study [115] are given in the last row. References for literature values: ASAs & DAs [112–115]; BCs [112, 114, 115]; CAVs [112–115]. (PDF) [file pcbi.1011496.s046.pdf]

Supporting Tables.

S29 Table

	SAs: WSS [dyne/cm^2]	DAs: WSS [dyne/cm^2]	Cs: WSS [dyne/cm^2]	AVs: WSS [dyne/cm^2]
C57BL/6_{I}	49.5 ± 67.8	28.7 ± 13.7	21.2 ± 45.0	5.7 ± 3.0
C57BL/6_{II}	51.5 ± 68.8	29.4 ± 13.6	44.1 ± 81.6	7.0 ± 3.1
BALB/c_{I}	47.3 ± 63.6	30.7 ± 15.0	41.8 ± 66.8	7.1 ± 3.3
BALB/c_{II}	46.1 ± 55.4	32.5 ± 15.1	62.9 ± 104.1	8.3 ± 2.9
Literature	8 - 150[A]	8 - 150[A]	5 - 70[B]	5 - 50[C]
